# An Enhancer-Based Analysis Revealed a New Function of Androgen Receptor in Tumor Cell Immune Evasion

**DOI:** 10.3389/fgene.2020.595550

**Published:** 2020-12-02

**Authors:** Yuan Wang, Jiajia Li, Jingjing Li, Peipei Li, Li Wang, Lijun Di

**Affiliations:** Cancer Center, Faculty of Health Sciences, University of Macau, Macau, China

**Keywords:** enhancer, GRO-seq, prostate cancer, androgen receptor, immune evading

## Abstract

Cancer is characterized by dysregulation at multiple levels, such as gene transcription. Enhancers are well-studied transcription regulators that can enhance target transcripts through DNA loop formation mediated by chromosome folding. The gain or loss of the interaction between an enhancer and its target gene has a critical effect on gene expression. In this study, we analyzed GRO-seq data to identify active enhancers from seven common cancer cell lines and studied the function of these enhancers across multiple cancer types. By constructing an “enhancer effect score” (EES), we found a significant correlation between EES and tumor-infiltrating lymphocytes (TILs) in prostate cancer. Further analysis revealed that androgen receptor (AR) plays an important role in regulating the immune checkpoint gene PVR via its enhancer. These results suggest that AR contributes to prostate cancer aggressiveness by promoting cancer cell immune evasion.

## Introduction

Cancer is one of the leading causes of death. Compared with normal cells, cancer cells possess some common features, such as genome instability and unlimited replicative potential, which are known as cancer hallmarks (Hanahan and Weinberg, 2000, 2011). The emergence of these cancer hallmarks is driven by mutations accumulated during the process, in which normal cells transform tissue to states from hyperplasia to dysplasia until neoplasia is established. In addition, large-scale genome-wide studies (GWASs) of cancer patients have indicated that dysregulation of gene expression significantly contributes to cancer development (Sarkar et al., 2013).

In human cells, the protein-coding genes account for only 2% of the genome, and a rough estimation predicts that no more than 40,000 genes, at maximum, are encoded. In addition, recent studies revealed that a significant proportion of the remaining 98% of genomic DNA, which is non-coding, participates in the regulation of gene expression. Some of the relatively conserved DNA sequences serve as either cis or trans regulatory elements but do not encode proteins. Enhancers are well-characterized regulatory elements and contribute mainly to positive gene expression by interacting with gene promoters and increasing the efficiency of RNA polymerase II recruitment and engagement (Koch et al., 2011). Thus, the importance of enhancers has been demonstrated in many important biological processes, such as cancer development, cell identity formation (Whyte et al., 2013), and cell fate determination (Adam et al., 2015). During cancer development, enhancers play important roles in driving oncogene transcription and may determine some cell-specific traits (Gonen et al., 2018). Enhancers are hotspots enriched with binding motifs for transcriptional factors (TFs) that confer enhancer–gene (EG) specificity. A prevalent model depicts both gene-specific TFs and some common TFs, such as mediator complexes, as participants in the establishment of cross talk between enhancers and their target genes (Soutourina, 2019). Any variation in the enhancer locus that breaks the binding affinity for TFs results in abnormal gene expression, which can be correlated with cancer development (Sur and Taipale, 2016).

Several high-throughput next-generation sequencing (NGS)-based technologies have been developed to characterize enhancers based on common characteristics, such as ChIP-seq analysis of H3K4me1 (Rada-Iglesias et al., 2011), H3K27ac (Creyghton et al., 2010), and P300 (Visel et al., 2009; May et al., 2011); DNase-seq (Bernstein et al., 2010); FAIRE-seq (Giresi et al., 2007); and ATAC-seq (Corces et al., 2018). More recently, enhancer locus-specific transcription activity was captured and revealed the positions of active enhancers. These transcripts are termed “eRNAs.” Most eRNAs are not stable and lack a poly-A tail, which make them difficult to capture by traditional RNA-sequencing methods. Thus, a series of novel technologies, such as GRO-seq (Melgar et al., 2011), PRO-seq (Kwak et al., 2013), and NET-seq (Mayer et al., 2015), were designed to capture and characterize eRNAs and enhancers. GRO-seq is the most widely applied technology to capture eRNAs because it is based on the capture of newly synthesized RNA after transcriptional activity is restarted in an isolated nucleus, which is also known as “nuclear run-on.” Transcripts mapping the intergenic region were recognized as potential eRNAs identified in enhancer regions (Liu et al., 2017; Nagari et al., 2017; Franco et al., 2018).

In this paper, we systematically characterize the active enhancers in several cancer cells based on eRNA transcription as characterized by GRO-seq. The significant EG pairs were further characterized based on the analysis of the activity of these enhancers and the transcription activity of genes in pan-cancer samples. This large-scale analysis of coordinated enhancer and gene expression allowed us to identify an enhancer-dominated cancer hallmark, “evading immune detection,” in prostate cancer. Our data indicate that the characterization of enhancers based on eRNA is a robust way to investigate the important functions of enhancers in cancer.

## Materials and Methods

### Data Collection

GRO-seq data for cell lines of A549, MCF7, HCT116, HeLa, HepG2, LNCap, and SKOV3 were collected from Gene Expression Omnibus (GEO) database. H3K4me1, H3K27ac ChIP-seq, and DNase-seq broad peak files for these cell lines were collected from both GEO and ENCODE. As the H3K4me1 ChIP-seq data are not available, SKOV3 is skipped for enhancer marker enrichment analysis. And DNase-seq for SKOV3 ovarian cancer was from ENCODE ENCSR712PYJ, a dataset from a 30-year-old female adult ovary. The detail of data accession can be found in Supplementary Table 1. ATAC-seq data for relative cancer types of these cell lines were collected from the work of M. Ryan (Corces et al., 2018), which detected 23 cancers from The Cancer Genome Atlas (TCGA). The peak files were used as opening chromatin regions and compared with GRO-seq defined active transcript regions. And the bigwig files were used to calculate the ATAC signal on GRO-seq defined enhancers. CrossMap was used to transfer the hg38 to hg19, and bigWigAverageOverBed from UCSC (University of California, Santa Cruz) tools was used to count the reads on enhancers. The established enhancers were from four different data resources: (1) FANTOM5, (2) dbSUPER, (3) VISTA, and (4) Ensembl. All the data were collected in or transformed to hg19 reference with UCSC Lift Over tools. Tumor immune cell infiltration data were download from The Cancer Immunome Atlas (TCIA)^1^ and Tumor IMmune Estimation Resource (TIMER)^2^ database. TCIA used normalized enrichment score (NES) to represent 28 immune cell infiltration, while TIMER was based on gene expression, which related with tumor purity to estimate B cell, CD4+ T cell, CD8+ T cell, neutrophil, macrophage, and dendritic cell infiltration. Androgen receptor (AR) ChIP-seq in prostate cancer and normal samples was downloaded from GSE56288. The processed .bw files were used to generate Supplementary Figure 17 with Integrative Genomics Viewer (IGV). ChIA-PET data of MCF7 were download from ENCODE ENCSR000CAA dataset. Genes that belong to each cancer hallmark were download from Gene Ontology, which were reported to be related with cancer hallmarks (Plaisier et al., 2012). Data for validating AR effect on enhancer PVR regulation are from GSE117193 and GSE120720.

### Data Procession

**FIGURE 1:**
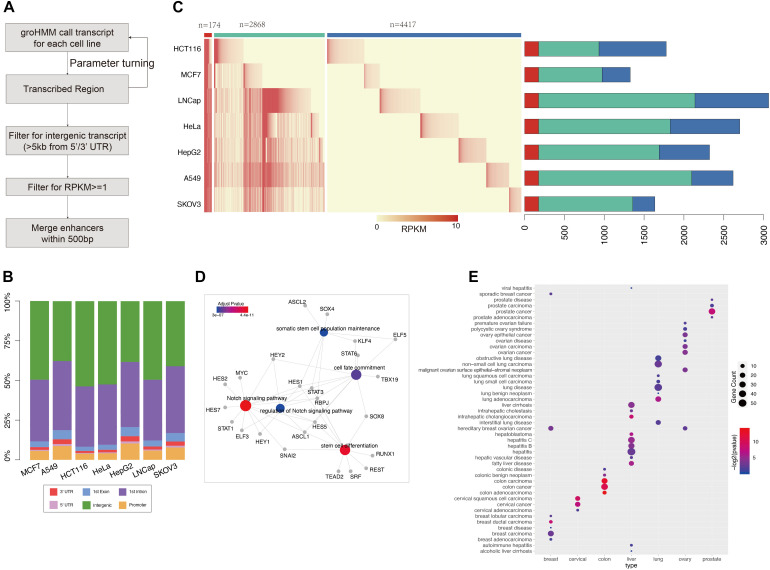
GRO-seq defined common and cancer-seecific enhancer sets **(A)** pipeline for enhancer prediction with GRO-seq data. **(B)** The genome annotation for groHMM produced “transcribed” regions (output for the second step in figure **A**). Different color shows the transcripts belong to each class as labeled. The y-axis points out the proportion for each class, **(C)** the left heatmap shows the expression level of each enhancer in each cancer call line. The color ber on the top represent common enhencer (red), intermediate enhancer (green), and cancer-specific enhancer (blue). The barploat on the right shows the percentage of each enhancer class in total enhancer sets of every cancer type. **(D)** The gene ontology analysis performed with transciption factors binding on common enhancers which is predicted by MEME suite. The figure was generated by “clusterprofilter” R package. The colored nodes are function GO term and gray dots show genes belong to each GO term. The color represents enrichment sighnificant *p*-value. **(E)** Disease Ontology (DO) analysis results for cancer-specific enhancer target genes. The nodes color represents enrichment significant and size shows number of target enriched in each disease related gene set.

For GRO-seq, data were mapped to hg19 reference with SOAP-2.21 using the parameter −r 0 −v 3 −n 10 −l 32. The mapped data were used to call active transcribed regions with R package groHMM. The parameter turning step was executed to give the optimal parameters. The details of the cancer type, relative cell line, parameter for groHMM, and data performance are listed in Supplementary Table 1 and Figure 1. For ChIP-seq, data were mapped to hg19 with bowtie2 with a default parameter, and the mapped reads with quality no less than 30 were filtered.

### Enhancer Definition

The pipeline groHMM was performed to call active transcripts for each cell type, average RPKM ≥ 1 was used as cutoff for cell types with replicates, and RPKM ≥ 2 was used for those without replicates to filter out active transcripts with low confidence. In order to avoid the interference from coding genes, the transcripts were filtered with bedtools suite intersect −v function to include the ones 5 kb away from any refseq genes 5′/3′-UTR regions. The refseq genes were downloaded from UCSC table with reference hg19. And then, the filtered transcripts were combined to obtain new transcript boundary across all cell types by merging the transcripts within 500 bp (Danko et al., 2015), and the merged transcripts were taken as putative enhancers for the following analysis. Functional enhancers were defined as enhancers that show significant Pearson correlation with target genes in any cancer types (adjusted *p*-value < 0.05).

### Enhancer Target Assignment

The genes within 500 kb were considered as enhancer targets at first. In case of missing long-distance pairs, enhancer-target pairs defined by GeneHancer database were also included. Then Pearson correlation was calculated with TCGA data to confirm the regulation relationship between each pair. Three types of data were used to estimate enhancer activity in each TCGA sample: (1) for TCGA ATAC-seq data, the enhancer activity was measured by average ATAC-seq signal on enhancer regions. (2) For TCGA RNA-seq data, as Han Liang et al. have published the enhancer expression profile for TCGA samples (Chen et al., 2018), we simply take their established expression value as our enhancer activity if their defined enhancer is situated within 500 bp of our enhancers. (3) For TCGA methylation data, the average methylation beta value from methylation 450 K array was used to represent our enhancer activity. The Pearson correlation based on these three datasets were calculated, and false discovery rate (FDR) <0.05 accompanied with no less than 0.3 absolute correlation value was used as cutoff to filter confident results.

### Enhancer Effect Score

The correlation of EG pairs reflects the regulation ability of enhancer to target genes. To quantify the enhancer regulation of a set of genes, such as a set of genes that belong to the same pathway, the average of correlations weighted by target gene expression was used. As limited by dataset, not all the genes were included in our EG pairs. Therefore, hypergeometric distribution test was performed to calculate the enrichment of target genes in specific gene set, and the *p*-value was used to adjust the average correlation. Finally, this score presents the enhancer regulation to specific pathways and we named it as enhancer effect score (EES), with the formula as follows:

$${\text{EES}}_{s}=\left(-{\text{log}}_{2}\,P-{\mathrm{value}}_{\text{enrichment}}\right)*\frac{\sum_{i}^{n}{\text{RPKM}}_{i}*{\left|{\text{Cor}}_{\text{max}}\right|}_{i}}{\sum_{i}^{n}{\text{RPKM}}_{i}}$$

*P*-value_*enrichment*_ is calculated by hypergeometric distribution; n is the total number of genes that belong to a specific gene set; RPKM*_*i*_* is the gene i expression in sample s; and Cor_*max*_ is the Pearson correlation coefficient between gene *i* and its enhancer, when gene *i* is correlated with more than one enhancer, and the max value was used.

### Motif Enrichment Analysis

MEME-ChIP was used to detect the binding TF enriched on 174 common active enhancers. We extend 50 bp on both sides of each enhancers and sliced enhancers into 100-bp-long pieces to make sure that every part of the enhancer is under motif searching area. TOMTOM was selected to match the motif to known TFs. Gene Ontology analysis was performed on these enriched TFs with R package “clusterProfiler.”

### Modulator Searching

The samples were sorted according to certain gene expression. The first and last quarters were selected as low and high expressed groups, respectively. The correlation was recalculated within these two groups; the difference between two correlation value (Diffcor) was used to filter modulators. The genes with Diffcor of no less than 0.5 were considered related with enhancer regulation to PVR expression.

### Statistical Analysis

Differentially expressed genes were defined as fold change larger than 1.5 and *t*-test FDR < 0.05. For those cancer types that have no normal data or less than five normal samples, the average expression from all TCGA normal samples was used to compare with that of tumors. The H3K27ac and H3K4me1 enrichment on enhancer regions was performed with deepTools software. The overlap of opening chromatin regions defined by GRO-seq, ATAC-seq, and DNase-seq was done by “findOverlapsOfPeaks” functions in “ChIPpeakAnno” package. The conservation score was measured with phastCons score obtained by “phastCons100way.UCSC.hg19” package. And the average phastCons score was used to calculate enhancers and their 2.5-kb flanking region with 50 bp as slicing window. And for the genome background, we randomly select the same number of regions with functional enhancers, also with the same length as average of these enhancers, and we use the same method to calculate the phastCons distribution around these random regions.

## Results

### GRO-Seq Revealed Common and Specific Enhancers Across Cell Lines

To study data on active enhancer behavior in cancer, GRO-seq data for seven common cancers (LUAD, BRCA, CESC, COAD, LIHC, PRAD, and OV) were collected from the GEO database. The GroHMM method was performed on these GRO-seq data (Figure 1A), and the active transcribed regions were identified. These regions were considered open chromatin regions, and potential enhancers were further extracted from them. On average, 86% (ranging at 79% for the HepG2 samples and 91.7% for the HCT116 samples) of these active transcribed regions were located in the non-coding region (Figure 1B). All of these transcribed regions were compared with results from analyses of DNase-seq and ATAC-seq, which are two widely used methods for detecting opening chromatin regions (Roadmap Epigenomics Consortium, Kundaje et al., 2015; Corces et al., 2018). GRO-seq can cover more than 87% peaks obtained from ATAC-seq, and approximately 92% peaks were obtained from DNase-seq. In addition, GRO-seq defined 435,838 active transcribed regions that were covered by either of the two other methods (Supplementary Figure 2). These results show that GRO-seq is more sensitive in predicting regions with an open chromatin status.

Following the pipeline presented in Figure 1A, GRO-seq-defined active transcribed regions situated outside 5 kb of the coding gene were obtained by filtering. The number of GRO-seq-defined “transcribed” regions is related to sequencing depth (Pearson correlation: 0.83) (Figure 1 and Supplementary Figure 3); for example, HeLa cells contain three-fold more active transcripts than HepG2 cells. Thus, to obtain more reliable enhancer sets, a cutoff of RPKM ≥ 1 was used (see section “Materials and Methods”) to filter the enhancer sets. Finally, 7,459 putative enhancers were identified. More than one-half of these enhancers (4,417/7,459) show activity in only one cancer type, and we named them “cancer-specific enhancers.” However, 174 enhancers showed consistent eRNA expression across all the cancer types assessed, and we defined these enhancers as “common enhancers” (Figure 1C). These 174 common enhancers were bound by TFs related to common cancer features, such as “Notch signaling pathway” and “stem cell differentiation” (Figure 1D). “Notch signaling” is pivotal for cell fate determination, and its dysregulation was reported to be involved in oncogenesis (Allenspach et al., 2002). Additionally, cancer stem cells are known to contribute to cancer initiation and progression (Yu et al., 2012; Venkatesh et al., 2018). The correlation analysis of the common enhancers and the expression of their downstream target genes support the hypotheses that the common enhancers contribute to the activation of these pathways and tumor progression. The cell type-specific enhancers were also enriched with corresponding cancer genes according to a disease ontology analysis (Figure 1E).

GRO-seq-defined enhancers showed considerable enrichment with H3K27ac and H3K4me1 (Supplementary Figure 4). Another four enhancer resources (Ensembl, dbSUPER, FANTOM5, and VISTA) were also accessed to further characterize the GRO-seq-defined enhancer sets, and approximately 41% of the GRO-seq-defined enhancers were identified in at least one of these databases (Supplementary Figure 5). A comparison of enhancer markers among these enhancer sets was also performed, and GRO-seq-defined enhancers were similarly enriched with these markers, suggesting that GRO-seq is a reliable approach to explore the function of enhancers in cancer (Supplementary Figure 6).

### Enhancer-Based Analysis Revealed Cancer Risk Regulatory Events

**FIGURE 2:**
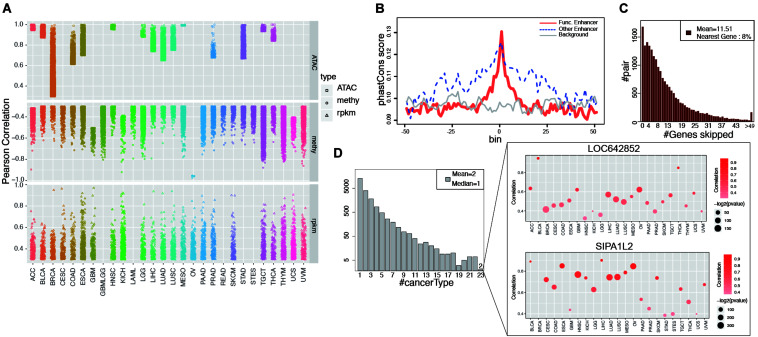
Functional enhancers and cancer common EG pairs were found by correlation analysis. **(A)** Shows all the significant EG pairs identified with ATAC-seq, RNA-seq and DNA methylation data. Each dot denotes one EG pair and different shape was used to indicate different data types. Different color was assigned to different cancer types. **(B)** *Y*-axis is the average phastCons value for each bin. The lines indicate the phastCons value on functional enhancers (red, whose activity show significant correlation with target gene expression), other enhancers (blue, which defined by GRO-seq but show no significant correlation with target gene expression) and genome random selected regions (gray, with have similar length with functional enhancers) and their flanking 2.5 kb regions. **(C)** The height of each bar shows the number of EG pairs within which the target genes skip a certain number of genes (*x*-axis) and correlated with enhancer on the distal region. **(D)** The left barplot shows the number of EG pairs that display significant correlation in a certain number of cancer types (*x*-axis). The right panel show the detail of two EG pairs which show correlation in 23 cancers. The color of dot indicates correlation and the size represents *p*-value from Pearson Correlation analysis.

To identify the regulatory targets of the identified enhancers, enhancers and genes within 500 kb were used to comprise EG pairs (Javierre et al., 2016; Corces et al., 2018), and together with the EG pairs from the GeneHancer database, more than 84,000 pairs were collected. Although the enhancers were mainly retrieved from the GRO-seq data based on seven types of cancer cells representative of seven major types of cancer, a significant fraction of these enhancers are active enhancers in more than two types of cancer (from 52.5% in the HCT116 cells to 83% in the SKOV3 cells), suggesting that different types of cancer share enhancers to a certain extent. Hence, in addition to the seven common cancers, more cancer types were considered in the following study of the regulatory relationships between enhancers and genes. To confirm the regulatory relationship, the Pearson correlation between enhancer activity and gene expression was calculated for each established EG pair, within which the enhancer activity was determined by ATAC-seq, DNA methylation, or RNA-seq according to TCGA data (see section Materials and Methods). In total, 20,930 EG pairs comprising 4,815 enhancers and 10,132 genes showed a significant correlation between enhancer activity and gene expression (Figure 2A). The correlation results from the ATAC-seq, DNA methylation, and RNA-seq data were combined, and 75% of the EG pairs were supported by the ATAC-seq (39 and 7% of the findings were supported by the methylation and RNA-seq data, respectively), and 386 EG pairs were supported by all three types of data (Supplementary Figure 7A).

The enhancers that showed a significant correlation with target gene expression were considered “functional enhancers.” Consistent with established knowledge, functional enhancers are more conserved than flanking regions and random genomic backgrounds (Figure 2B). Consistent with a previous report (Javierre et al., 2016), when only EG pairs from GeneHancer were taken into account, 96% of the targets were within 500 kb of enhancers (Supplementary Figure 7B). More than one-half of target gene expression is regulated by at least two enhancers, whereas on average, one enhancer regulates four different targets (Supplementary Figures 7C,D). Intermediate DNA loop generation is a known mechanism that enables the direct interaction between an enhancer and a promoter (Su et al., 1991). ChIA-PET has been applied to study the long-distance interaction between an enhancer and a promoter mediated by TF or Pol II. We analyzed the Pol II ChIA-PET data for MCF-7 cells and observed that the EG pairs with DNA loops showed a higher correlation than those without loops (Supplementary Figure 7E). Unexpectedly, among all the target genes we analyzed, only 8% of the enhancer target genes were located in neighboring regions (Figure 2C). Previous reports showing that the nearest genes are more likely to be enhancer targets (Heintzman et al., 2009; Visel et al., 2009; Xu et al., 2012); however, our findings suggest that enhancers tend to skip the nearest genes, which is consistent with the result by Sanyal et al. (2012).

In our study, among all 20,930 EG pairs, 70% showed a significant correlation in at least two cancer types. Two EG pairs containing the target genes SIPA1L2 (signal-induced proliferation-associated 1 like 2) and LOC642852 showed a significant correlation in 23 cancers (Figure 2D). SIPA1L2 is related to GTPase activator activity and the Ras signaling pathway. The dysregulation of SIPA1L2 is also known as a risk factor for cancer. Although a non-coding gene, LOC642852 is widely expressed in different cancers, and some studies have reported that LOC642852 is related to colorectal cancer and pancreatic cancer (Giulietti et al., 2018; Zhang et al., 2019).

### Enhancers Contribute to Cancer Signaling Pathways

**FIGURE 3:**
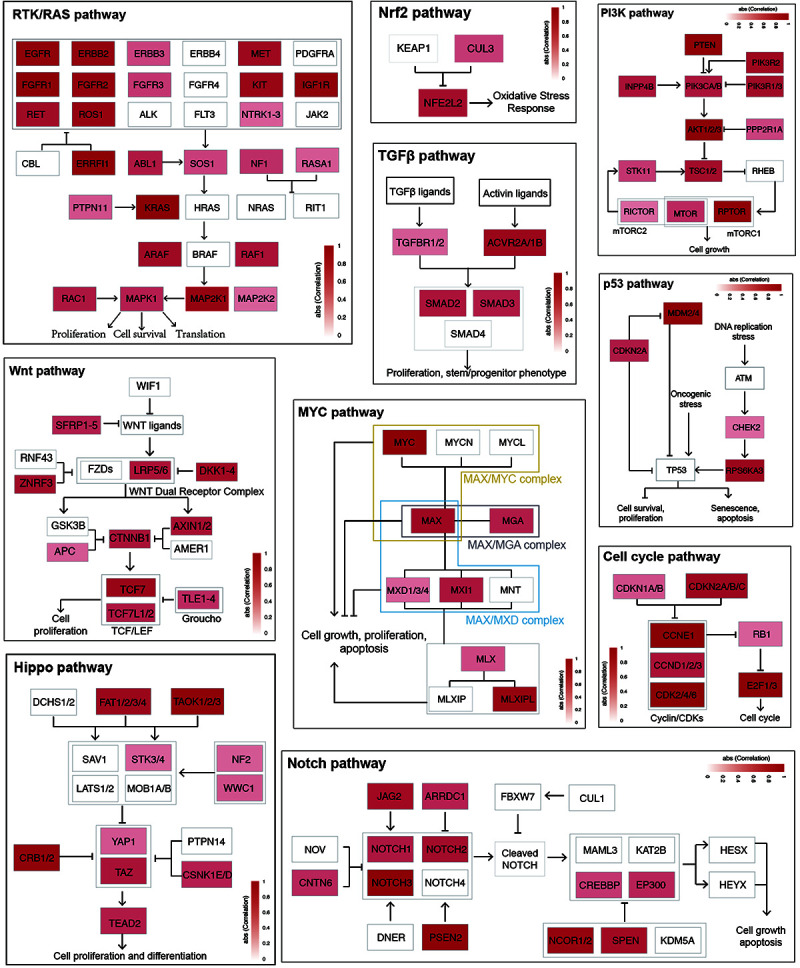
Enhancer effects in establishing cancer signaling environments. The genes within each cancer signaling pathway were colored according to the max correlation across all cancer types.

**FIGURE 4:**
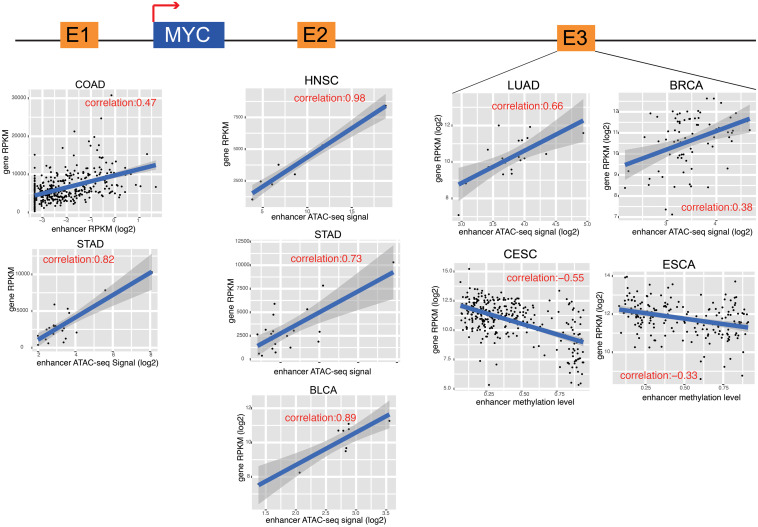
MYC and its enhancers in different cancers. The cartoon shows the relative position of MYC and its enhancers. The detail of correlation results was showed in the down panel. Each dot represents one cancer samples.

To further characterize enhancer functions in cancer, data on 174 genes in 10 common oncogenic signaling pathways were collected (Sanchez-Vega et al., 2018). More than one-half of the genes in each pathway were significantly correlated with enhancer activity, which indicates the important function of enhancers in regulating the activity of these pathways (Figure 3 and Supplementary Figure 8). For example, MYC is a key oncogene in the MYC signaling pathway, and the dysregulation of MYC is related to the development of many cancers (Boxer and Dang, 2001; Eilers and Eisenman, 2008; Gabay et al., 2014). Three different enhancers of MYC were identified (Figure 4). MYC is highly expressed in many cancer types, but the abnormal activation of MYC is related to different enhancers (Lancho and Herranz, 2018). In COAD and STAD, MYC expression is related to the enhancer located approximately 160 kb upstream of the MYC promoter (E1), while for HNSC and BLCA, the expression of MYC is correlated with the enhancer located near the promoter downstream (E2). Interestingly, MYC expression is significantly correlated with both of these enhancers in STAD. To study how these two enhancers contribute to MYC expression in STAD, both one-way and multivariate analyses of variance were performed, and the results showed that E1 contributes to MYC more significantly (Supplementary Figure 9). Surprisingly, low expression of both E1 and E2 results in the recovery of MYC expression in STAD, suggesting that other unknown regulatory mechanisms might be involved. The third enhancer (E3) of MYC is located far downstream from the gene, and some other genes are in the intermediate region. However, the activity of this enhancer only shows a strong correlation with MYC expression in LUAD, BRCA, CESC, and ESCA. Interestingly, the single-nucleotide polymorphism (SNP) “rs11780156,” which has been reported as a risk SNP in GWAS in breast cancer was found in the MYC enhancer E3 (Shi et al., 2016). Moreover, several other SNPs (rs6999335, rs6999897, rs11783807, rs56152647, rs6992491, and rs67397162), in linkage disequilibrium with SNP “rs11780156,” are all located in this enhancer according to HaploReg (Ward and Kellis, 2012), suggesting that E3 plays an important role in increasing the risk of BRCA.

### Enhancers Regulate Cancer Hallmarks

**FIGURE 5:**
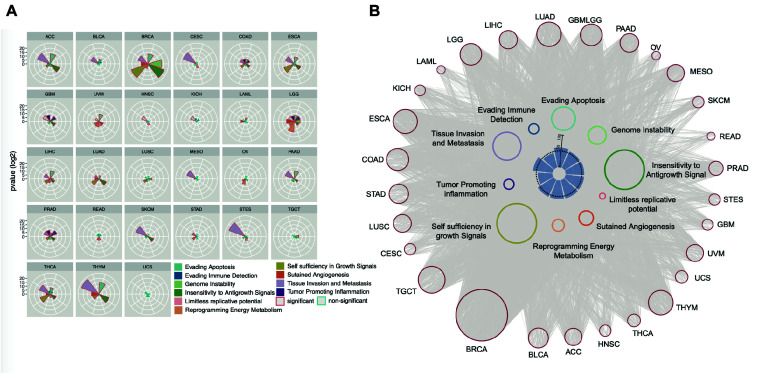
Enhancer regulates cancer hallmark. **(A)** Enhancer target genes in each cancer types were used to calculate enrichment in every cancer hallmark by Hypergeometric distribution test. The significant enriched hallmark was labeled with red border. **(B)** The EG pairs within each cancer hallmark were shown as network. The red dots in outer circle were enhancers in each cancer and the colored dots in inner circle were the genes belong to every hallmark. The lines between enhancers and hallmark genes were decided by correlation analysis results. The inner barplot indicates the percentage of enhancer targets take in total hallmark genes. The 50% were marked with dash line.

We also examined the enrichment of enhancer target genes in cancer hallmarks (Hanahan and Weinberg, 2011). As expected, the target genes were significantly enriched in these 10 hallmarks (Figure 5A). For example, the hallmark of tissue invasion and metastasis is enriched in nearly all tumor types, suggesting that the genes that promote tumor invasion and metastasis are most likely the regulatory targets of active enhancers. Some other hallmarks are enriched only in a few types of cancer, suggesting that while some common traits are shared by most types of cancer, the heterogeneity of cancer hallmarks can be used to distinguish different types of cancer. Although the enrichment patterns are different among cancer types, approximately 50% of genes belonging to each cancer hallmark were found to be regulated by enhancers (Figure 5B), suggesting that enhancers play important roles in characterizing cancer traits.

### Tumor-Infiltrating Lymphocytes Are Regulated by Enhancers

**FIGURE 6:**
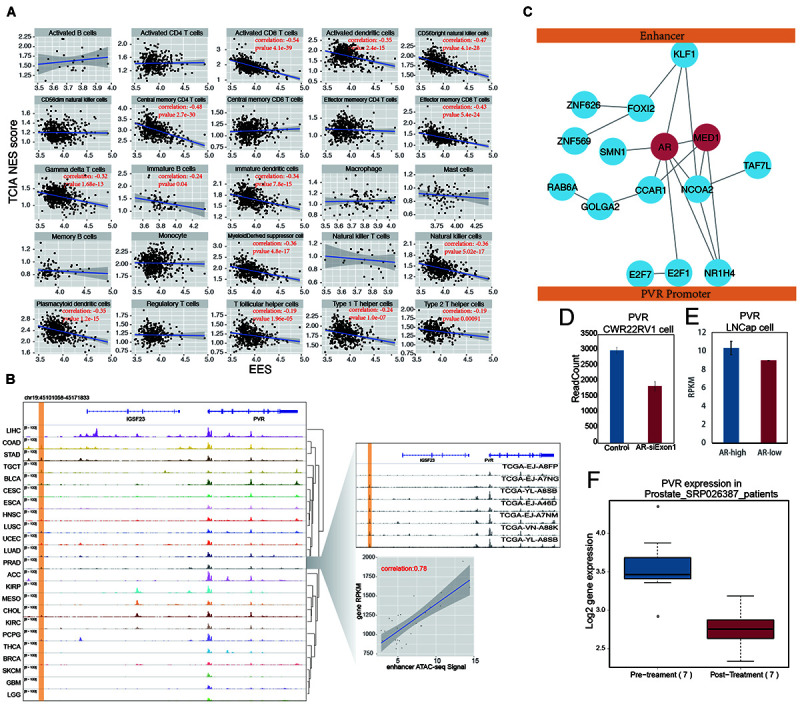
AR promote tumor immune evading by binding on enhancer region and stimulate PVR expression. **(A)** The correlation between EES score and TILs score measured by TCIA database in prostate cancer. Each dot indicates one prostate samples. The significant correlation (*p*-value < 0.05) were labeled in red. **(B)** The ATAC-seq signal on PVR and its enhancer locus. The PVR enhancer locus were labeled with yellow shading. The detail of PVR expression and enhancer activity correlation was shown in the right down panel. Each dot represents one prostate cancer sample. The right up panel was ATAC-seq signal on PVR locus in random selected seven prostate samples. **(C)** The cartoon for artificial protein complex predicted by combination results from DNA motif analysis, modulator searching analysis and protein-protein interaction network (from STRING database). MED1 and AR were labeled in red. **(D)** The PVR expression was shown in barplot between group of prostate cancer cell line (CWR22RV1) with (red) and without (blue) AR knockdown in the first exon. **(E)** The PVR expression were shown in barplot between groups of prostate cancer cell line (LNCap) with higher AR level (blue) and lower AR level (red). **(F)** The PVR expression was shown in boxplot between prostate samples before and after Androgen Ablation therapy.

A significant number of genes identified in our EG list were involved in one of the important cancer hallmarks, the “evading immune detection” (EID) pathway in PRAD, COAD, and GBMLGG cancers, among which PRAD shows the most significant enrichment (Figure 5A). These three cancer types generally have higher tumor purity than the other types (Supplementary Figure 10). To evaluate the effect of an enhancer on the tumor immune response, an EES was developed. EES is a weighted average Pearson correlation calculated for all EG pairs within an EID pathway. We found that, in PRAD, the EES was significantly correlated with tissue infiltration of many immune cells, such as natural killer cells, activated CD8+ T cells, dendritic cells, T helper cells, and immature B cells (Figure 6A and Supplementary Figure 11). However, the activity of the EID pathway, which was measured by the average gene expression, was not related to tumor-infiltrating lymphocytes (TILs) (Supplementary Figures 12A,B). Since each enhancer may regulate more than one gene, depending on the local chromatin organization, this result suggests that enhancers can be better representatives of local genomic activity than any single gene.

By examining every EG pair within the EID pathway, we found that PVR and its enhancer showed the most significant correlation (Supplementary Figure 13A). PVR, also known as CD155, is an immune checkpoint that can suppress the immune response through its interaction with the T-cell receptor TIGIT (Inozume et al., 2015). PVR is highly expressed in many cancers, including prostate cancer (Supplementary Figure 13B), and its high expression is associated with poor clinical outcomes for prostate cancer patients (Supplementary Figure 13C). The enhancer of PVR identified in this study was situated approximately 40 kb upstream of the promoter, and the correlation between the enhancer activity and gene expression was 0.78; PVR is actively transcribed in many cancers (Figure 6B), and the putative enhancer of PVR was found to be positively correlated with PVR gene expression across all examined cancer types (Supplementary Figure 14). The activation of this enhancer is accompanied by PVR expressed in many cancers, such as ESCA, HNSC, and LUSC (Figure 6B). In other cancer types, such as KIRP, MESO, and CHOL, PVR expression also seems to be related to a putative intergenic enhancer in an intron of IGSF23.

The regulation of the target genes by enhancers is influenced not only by enhancer activity but also by factors mediating the interactions between the enhancer and the promoter. Since MED1 is an essential factor for enhancer regulation of the target gene (Sabari et al., 2018; Zamudio et al., 2019), the influence of MED1 on the correlation between the enhancer and PVR expression has been validated (Supplementary Figure 15). To globally explore the factors that potentially mediate the interaction between the PVR enhancer and promoter, a search for modulators was performed. Highly expressed genes contributing to the high correlation of the PVR enhancer and promoter were considered potential modulators, and 1,215 PVR modulators were identified by this method (Supplementary Figure 16). The top 20 modulators were selected for further characterization, and among these candidate, proteins directly binding to the PVR enhancer and promoter were identified by motif screening. The 20 modulators found in this manner were further filtered according to the STRING database. The proteins showing no connection to other modulators were removed, and the remaining modulators were used to form a protein–protein interaction network to predict the protein complex(es) mediating the interaction between the PVR enhancer and its promoter (Figure 6C). Within this predicted protein complex, in addition to MED1, AR was found to be an important hub node.

In support of our proposition, a reanalysis of AR binding with an AR ChIP-seq dataset containing both normal and tumor samples of prostate cancer revealed that AR binds only to the PVR enhancer region in the prostate tumor samples but not in the normal prostate tissue (Pomerantz et al., 2015). This result suggests that highly expressed PVR in tumors may correlate with the binding of AR to enhancers (Supplementary Figure 17). Moreover, the knockdown of AR in CWR22Rv1 prostate cancer cell lines led to decreased PVR expression (Hepburn et al., 2019; Figure 6D). PVR expression is also significantly higher in prostate cancer cells with high AR levels (Lee et al., 2019; Figure 6E). These results suggest that AR mediates PVR expression in prostate cancer. AR binds to androgen and then enters the nucleus to regulate gene transcription. The function of AR is affected when androgen levels are modulated. We generated a clinical dataset (Rajan et al., 2014) based on prostate cancer patients who had accepted androgen ablation therapy, and we found a significant decrease in PVR expression in patients after treatment (Figure 6F). Together, these results indicate that AR plays potential roles in regulating PVR expression by mediating its promoter interaction with a distal enhancer.

## Discussion

Enhancers are critical regulatory elements of gene expression, and the characterization of the dysregulation of enhancers is important in cancer research (Kron et al., 2014; Yuan et al., 2017). In this study, we analyzed GRO-seq data of cancer cell lines to globally identify enhancers based on eRNA expression. The importance of these enhancers was further investigated in cancer databases, and the results revealed that these enhancers are important for regulating cancer signaling pathways and cancer hallmarks. Among the enhancers, a critical enhancer of the PVR gene was investigated in prostate cancer owing to its potential regulatory role in mediating immune repression. Interestingly, AR was found to be the trans-regulator of this enhancer and to be positively correlated with highly expressed PVR. As a risk factor for prostate cancer, AR has been targeted in clinical treatment for many years (Huggins and Hodges, 1972). Previous studies have mainly focused on AR as a type of nuclear receptor TF that has the ability to promote tumor growth and development by binding to downstream target genes and mediating the activation of genes related to proliferation, anti-apoptosis, or other cancer genes (Huang et al., 2018). Our results, on the other hand, suggest that AR binds to the enhancer region and amplifies the transcription of PVR genes, which results in the suppression of immune cells. This novel finding not only provides new clues to a new target gene regulated by AR but also indicates a previously unknown working mechanism of AR; that is, it binds to gene enhancers. In fact, AR binding to enhancers has been previously reported, suggesting that regulating gene expression via its enhancer is one of the important working mechanisms of AR.

The initial efforts in characterizing enhancers were based on DNase I hypersensitivity. With the accumulation of increased knowledge of enhancers, more features can be used to characterize them. The characterization of enhancers by quantitating eRNA is the newest approach and has shown remarkable robustness and sensitivity (Hah et al., 2011; Li et al., 2013). However, most eRNAs are not stable and require that the signal be captured through other approaches, such as GRO-seq. Since GRO-seq is more appropriately used to analyze cell lines than tumor tissues, we chose to reconcile the GRO-seq data obtained from cell lines with tumor gene expression data. While this approach enabled us to globally analyze how enhancers may influence gene expression and contribute to tumor traits, the nature of enhancers as dynamic regulatory elements limits the accuracy of this type of analysis. Some enhancers might be tumor specific, and some enhancers may be cell line specific. The reconciliation of the data obtained from different resources will greatly impede the identification of important EG pairs. In fact, when the depth of RNA-seq goes deeper, the potential to identify robust enhancers through characterized eRNA is increased. Thus, our pilot study suggests that eRNA is a reliable way to predict enhancers and has great power to identify important cancer driving enhancers. In the future, with eRNA data obtained directly from the sequencing data of tumor samples, more critical cancer-driving enhancers might be discovered.

Targeting the immune checkpoint is thus far the most successful tumor therapeutic approach. In addition to the well-recognized immune checkpoint blockers, such as CTLA-4 and PD-1, this type of immune blocker awaits for investigation of its involvement in cancer. PVR has been reported to be an entry receptor of poliovirus (Bowers et al., 2017). Binding of PVR to TIGIT, an inhibitory receptor, results in immune repression through the intracellular phosphorylation of substrates. Thus, high expression of PVR is associated with a worse outcome for several types of cancer (Nakai et al., 2010; Bevelacqua et al., 2012; Nishiwada et al., 2015). Our finding of a PVR enhancer is the first report indicating the potential role of PVR in prostate cancer. To our surprise, we were only able to observe that the EES of the EID pathway correlated with the outcome of patients. The correlation of the average expression of an EID pathway gene and the outcome for patients was not significant. However, we found that the PVR enhancer is in a very gene-rich region, suggesting that the PVR enhancer might also play roles in regulating the expression of other genes in addition to its dominant role as a PVR gene enhancer. In fact, regulating the expression of several genes by a single enhancer is not unusual. Thus, the EES of EID genes might be a better predictor of patient outcome than average gene expression.

## Data Availability Statement

The original contributions presented in the study are included in the article/Supplementary Material, further inquiries can be directed to the corresponding author/s.

## Author Contributions

The authors are grateful for all the support from lab members. LD helped to improve the project and manuscripts. JiaL, PL, and JinL provided technical support for analysis and manuscript assessment. All authors contributed to the article and approved the submitted version.

## Conflict of Interest

The authors declare that the research was conducted in the absence of any commercial or financial relationships that could be construed as a potential conflict of interest.

## Footnotes

1 https://tcia.at/home

2 https://cistrome.shinyapps.io/timer/

## References

[B1] Adam R. C. Yang H. Rockowitz S. Larsen S. B. Nikolova M. Oristian D. S. (2015). Pioneer factors govern super-enhancer dynamics in stem cell plasticity and lineage choice. *Nature* 521 366–370. 10.1038/nature14289 25799994 PMC4482136

[B2] Allenspach E. J. Maillard I. Aster J. C. Pear W. S. (2002). Notch signaling in cancer. *Cancer Biol. Ther.* 1 466–476. 12496471 10.4161/cbt.1.5.159

[B3] Bernstein B. E. Stamatoyannopoulos J. A. Costello J. F. Ren B. Milosavljevic A. Meissner A. (2010). The NIH roadmap epigenomics mapping consortium. *Nat. Biotechnol.* 28 1045–1048. 10.1038/nbt1010-1045 20944595 PMC3607281

[B4] Bevelacqua V. Bevelacqua Y. Candido S. Skarmoutsou E. Amoroso A. Guarneri C. (2012). Nectin like-5 overexpression correlates with the malignant phenotype in cutaneous melanoma. *Oncotarget* 3 882–892. 10.18632/oncotarget.594 22929570 PMC3478464

[B5] Bowers J. R. Readler J. M. Sharma P. Excoffon K. (2017). poliovirus receptor: more than a simple viral receptor. *Virus Res.* 242 1–6. 10.1016/j.virusres.2017.09.001 28870470 PMC5650920

[B6] Boxer L. M. Dang C. V. (2001). Translocations involving c-myc and c-myc function. *Oncogene* 20 5595–5610. 10.1038/sj.onc.1204595 11607812

[B7] Chen H. Li C. Peng X. Zhou Z. Weinstein J. N. Cancer Genome Atlas Research Network, (2018). A pan-cancer analysis of enhancer expression in nearly 9000 patient samples. *Cell* 173 386.e312–399.e312. 29625054 10.1016/j.cell.2018.03.027 PMC5890960

[B8] Corces M. R. Granja J. M. Shams S. Louie B. H. Seoane J. A. Zhou W. (2018). The chromatin accessibility landscape of primary human cancers. *Science* 362:eaav1898. 10.1126/science.aav1898 PMC6408149 30361341

[B9] Creyghton M. P. Cheng A. W. Welstead G. G. Kooistra T. Carey B. W. Steine E. J. (2010). Histone H3K27ac separates active from poised enhancers and predicts developmental state. *Proc. Natl. Acad. Sci. U.S.A.* 107 21931–21936. 10.1073/pnas.1016071107 21106759 PMC3003124

[B10] Danko C. G. Hyland S. L. Core L. J. Martins A. L. Waters C. T. Lee H. W. (2015). Identification of active transcriptional regulatory elements from GRO-seq data. *Nat. Methods* 12 433–438. 10.1038/nmeth.3329 25799441 PMC4507281

[B11] Eilers M. Eisenman R. N. (2008). Myc’s broad reach. *Genes Dev.* 22 2755–2766. 10.1101/gad.1712408 18923074 PMC2751281

[B12] Franco H. L. Nagari A. Malladi V. S. Li W. Xi Y. Richardson D. (2018). Enhancer transcription reveals subtype-specific gene expression programs controlling breast cancer pathogenesis. *Genome Res.* 28 159–170. 10.1101/gr.226019.117 29273624 PMC5793780

[B13] Gabay M. Li Y. Felsher D. W. (2014). MYC activation is a hallmark of cancer initiation and maintenance. *Cold Spring Harb. Perspect. Med.* 4:a014241. 10.1101/cshperspect.a014241 24890832 PMC4031954

[B14] Giresi P. G. Kim J. Mcdaniell R. M. Iyer V. R. Lieb J. D. (2007). FAIRE (Formaldehyde-Assisted Isolation of Regulatory Elements) isolates active regulatory elements from human chromatin. *Genome Res.* 17 877–885. 10.1101/gr.5533506 17179217 PMC1891346

[B15] Giulietti M. Righetti A. Principato G. Piva F. (2018). LncRNA co-expression network analysis reveals novel biomarkers for pancreatic cancer. *Carcinogenesis* 39 1016–1025. 10.1093/carcin/bgy069 29796634

[B16] Gonen N. Futtner C. R. Wood S. Garcia-Moreno S. A. Salamone I. M. Samson S. C. (2018). Sex reversal following deletion of a single distal enhancer of Sox9. *Science* 360 1469–1473. 10.1126/science.aas9408 29903884 PMC6034650

[B17] Hah N. Danko C. G. Core L. Waterfall J. J. Siepel A. Lis J. T. (2011). A rapid, extensive, and transient transcriptional response to estrogen signaling in breast cancer cells. *Cell* 145 622–634. 10.1016/j.cell.2011.03.042 21549415 PMC3099127

[B18] Hanahan D. Weinberg R. A. (2000). The hallmarks of cancer. *Cell* 100 57–70. 10647931 10.1016/s0092-8674(00)81683-9

[B19] Hanahan D. Weinberg R. A. (2011). Hallmarks of cancer: the next generation. *Cell* 144 646–674. 10.1016/j.cell.2011.02.013 21376230

[B20] Heintzman N. D. Hon G. C. Hawkins R. D. Kheradpour P. Stark A. Harp L. F. (2009). Histone modifications at human enhancers reflect global cell-type-specific gene expression. *Nature* 459 108–112. 10.1038/nature07829 19295514 PMC2910248

[B21] Hepburn A. C. Steele R. E. Veeratterapillay R. Wilson L. Kounatidou E. E. Barnard A. (2019). The induction of core pluripotency master regulators in cancers defines poor clinical outcomes and treatment resistance. *Oncogene* 38 4412–4424. 10.1038/s41388-019-0712-y 30742096 PMC6546609

[B22] Huang Y. Jiang X. Liang X. Jiang G. (2018). Molecular and cellular mechanisms of castration resistant prostate cancer. *Oncol. Lett.* 15 6063–6076. 29616091 10.3892/ol.2018.8123 PMC5876469

[B23] Huggins C. Hodges C. V. (1972). Studies on prostatic cancer. I. The effect of castration, of estrogen and androgen injection on serum phosphatases in metastatic carcinoma of the prostate. *CA Cancer J. Clin.* 22 232–240. 10.3322/canjclin.22.4.232 4625049

[B24] Inozume T. Yaguchi T. Furuta J. Harada K. Kawakami Y. Shimada S. (2015). Melanoma cells control anti-melanoma CTL responses via interaction between TIGIT and CD155 in the effector phase. *J. Invest. Dermatol.* 136 255–263. 10.1038/jid.2015.404 26763445

[B25] Javierre B. M. Burren O. S. Wilder S. P. Kreuzhuber R. Hill S. M. Sewitz S. (2016). Lineage-specific genome architecture links enhancers and non-coding disease variants to target gene promoters. *Cell* 167 1369.e1319–1384.e1319. 27863249 10.1016/j.cell.2016.09.037 PMC5123897

[B26] Koch F. Fenouil R. Gut M. Cauchy P. Albert T. K. Zacarias-Cabeza J. (2011). Transcription initiation platforms and GTF recruitment at tissue-specific enhancers and promoters. *Nat. Struct. Mol. Biol.* 18 956–963. 10.1038/nsmb.2085 21765417

[B27] Kron K. J. Bailey S. D. Lupien M. (2014). Enhancer alterations in cancer: a source for a cell identity crisis. *Genome Med.* 6:77. 10.1186/s13073-014-0077-3 PMC4254433 25473436

[B28] Kwak H. Fuda N. J. Core L. J. Lis J. T. (2013). Precise maps of RNA polymerase reveal how promoters direct initiation and pausing. *Science* 339 950–953. 10.1126/science.1229386 23430654 PMC3974810

[B29] Lancho O. Herranz D. (2018). The MYC enhancer-ome: long-range transcriptional regulation of MYC in cancer. *Trends Cancer* 4 810–822. 10.1016/j.trecan.2018.10.003 30470303 PMC6260942

[B30] Lee E. Wongvipat J. Choi D. Wang P. Lee Y. S. Zheng D. (2019). GREB1 amplifies androgen receptor output in human prostate cancer and contributes to antiandrogen resistance. *Elife* 8:e41913. 10.7554/eLife.41913 PMC6336405 30644358

[B31] Li W. Notani D. Ma Q. Tanasa B. Nunez E. Chen A. Y. (2013). Functional roles of enhancer RNAs for oestrogen-dependent transcriptional activation. *Nature* 498 516–520. 10.1038/nature12210 23728302 PMC3718886

[B32] Liu Y. Chen S. Wang S. Soares F. Fischer M. Meng F. (2017). Transcriptional landscape of the human cell cycle. *Proc. Natl. Acad. Sci. U.S.A.* 114 3473–3478. 28289232 10.1073/pnas.1617636114 PMC5380023

[B33] May D. Blow M. J. Kaplan T. Mcculley D. J. Jensen B. C. Akiyama J. A. (2011). Large-scale discovery of enhancers from human heart tissue. *Nat. Genet.* 44 89–93. 10.1038/ng.1006 22138689 PMC3246570

[B34] Mayer A. Di Iulio J. Maleri S. Eser U. Vierstra J. Reynolds A. (2015). Native elongating transcript sequencing reveals human transcriptional activity at nucleotide resolution. *Cell* 161 541–554. 10.1016/j.cell.2015.03.010 25910208 PMC4528962

[B35] Melgar M. F. Collins F. S. Sethupathy P. (2011). Discovery of active enhancers through bidirectional expression of short transcripts. *Genome Biol.* 12:R113. 10.1186/gb-2011-12-11-r113 PMC3334599 22082242

[B36] Nagari A. Murakami S. Malladi V. S. Kraus W. L. (2017). Computational Approaches for Mining GRO-Seq Data to Identify and Characterize Active Enhancers. *Methods Mol. Biol.* 1468 121–138. 10.1007/978-1-4939-4035-6_10 27662874 PMC5522910

[B37] Nakai R. Maniwa Y. Tanaka Y. Nishio W. Yoshimura M. Okita Y. (2010). Overexpression of Necl-5 correlates with unfavorable prognosis in patients with lung adenocarcinoma. *Cancer Sci.* 101 1326–1330. 10.1111/j.1349-7006.2010.01530.x 20331633 PMC11158505

[B38] Nishiwada S. Sho M. Yasuda S. Shimada K. Yamato I. Akahori T. (2015). Clinical significance of CD155 expression in human pancreatic cancer. *Anticancer. Res.* 35 2287–2297. 25862891

[B39] Plaisier C. L. Pan M. Baliga N. S. (2012). A miRNA-regulatory network explains how dysregulated miRNAs perturb oncogenic processes across diverse cancers. *Genome Res.* 22 2302–2314. 10.1101/gr.133991.111 22745231 PMC3483559

[B40] Pomerantz M. M. Li F. Takeda D. Y. Lenci R. Chonkar A. Chabot M. (2015). The androgen receptor cistrome is extensively reprogrammed in human prostate tumorigenesis. *Nat. Genet.* 47 1346–1351. 10.1038/ng.3419 26457646 PMC4707683

[B41] Rada-Iglesias A. Bajpai R. Swigut T. Brugmann S. A. Flynn R. A. Wysocka J. (2011). A unique chromatin signature uncovers early developmental enhancers in humans. *Nature* 470 279–283. 10.1038/nature09692 21160473 PMC4445674

[B42] Rajan P. Sudbery I. M. Villasevil M. E. Mui E. Fleming J. Davis M. (2014). Next-generation sequencing of advanced prostate cancer treated with androgen-deprivation therapy. *Eur. Urol.* 66 32–39. 10.1016/j.eururo.2013.08.011 24054872 PMC4062940

[B43] Roadmap Epigenomics Consortium, Kundaje A. Meuleman W. Ernst J. Bilenky M. Yen A. (2015). Integrative analysis of 111 reference human epigenomes. *Nature* 518 317–330. 25693563 10.1038/nature14248 PMC4530010

[B44] Sabari B. R. Dall’agnese A. Boija A. Klein I. A. Coffey E. L. Shrinivas K. (2018). Coactivator condensation at super-enhancers links phase separation and gene control. *Science* 361:eaar3958. 10.1126/science.aar3958 29930091 PMC6092193

[B45] Sanchez-Vega F. Mina M. Armenia J. Chatila W. K. Luna A. La K. C. (2018). Oncogenic signaling pathways in the cancer genome atlas. *Cell* 173 321.e310–337.e310. 29625050 10.1016/j.cell.2018.03.035 PMC6070353

[B46] Sanyal A. Lajoie B. R. Jain G. Dekker J. (2012). The long-range interaction landscape of gene promoters. *Nature* 489 109–113. 10.1038/nature11279 22955621 PMC3555147

[B47] Sarkar S. Horn G. Moulton K. Oza A. Byler S. Kokolus S. (2013). Cancer development, progression, and therapy: an epigenetic overview. *Int. J. Mol. Sci.* 14 21087–21113. 10.3390/ijms141021087 24152442 PMC3821660

[B48] Shi J. Zhang Y. Zheng W. Michailidou K. Ghoussaini M. Bolla M. K. (2016). Fine-scale mapping of 8q24 locus identifies multiple independent risk variants for breast cancer. *Int. J. Cancer* 139 1303–1317. 27087578 10.1002/ijc.30150 PMC5110427

[B49] Soutourina J. (2019). Mammalian mediator as a functional link between enhancers and promoters. *Cell* 178 1036–1038. 10.1016/j.cell.2019.07.040 31442397

[B50] Su W. Jackson S. Tjian R. Echols H. (1991). DNA looping between sites for transcriptional activation: self-association of DNA-bound Sp1. *Genes Dev.* 5 820–826. 10.1101/gad.5.5.820 1851121

[B51] Sur I. Taipale J. (2016). The role of enhancers in cancer. *Nat. Rev. Cancer* 16 483–493. 10.1038/nrc.2016.62 27364481

[B52] Venkatesh V. Nataraj R. Thangaraj G. S. Karthikeyan M. Gnanasekaran A. Kaginelli S. B. (2018). Targeting Notch signalling pathway of cancer stem cells. *Stem Cell Investig.* 5:5. 10.21037/sci.2018.02.02 29682512 PMC5897708

[B53] Visel A. Blow M. J. Li Z. Zhang T. Akiyama J. A. Holt A. (2009). ChIP-seq accurately predicts tissue-specific activity of enhancers. *Nature* 457 854–858. 10.1038/nature07730 19212405 PMC2745234

[B54] Ward L. D. Kellis M. (2012). HaploReg: a resource for exploring chromatin states, conservation, and regulatory motif alterations within sets of genetically linked variants. *Nucleic Acids Res.* 40 D930–D934. 22064851 10.1093/nar/gkr917 PMC3245002

[B55] Whyte W. A. Orlando D. A. Hnisz D. Abraham B. J. Lin C. Y. Kagey M. H. (2013). Master transcription factors and mediator establish super-enhancers at key cell identity genes. *Cell* 153 307–319. 10.1016/j.cell.2013.03.035 23582322 PMC3653129

[B56] Xu J. Shao Z. Glass K. Bauer D. E. Pinello L. Van Handel B. (2012). Combinatorial assembly of developmental stage-specific enhancers controls gene expression programs during human erythropoiesis. *Dev. Cell* 23 796–811. 10.1016/j.devcel.2012.09.003 23041383 PMC3477283

[B57] Yu Z. Pestell T. G. Lisanti M. P. Pestell R. G. (2012). Cancer stem cells. *Int. J. Biochem. Cell Biol.* 44 2144–2151. 22981632 10.1016/j.biocel.2012.08.022 PMC3496019

[B58] Yuan J. Jiang Y. Y. Mayakonda A. Huang M. Ding L. W. Lin H. (2017). Super-Enhancers Promote Transcriptional Dysregulation in Nasopharyngeal Carcinoma. *Cancer Res.* 77 6614–6626. 10.1158/0008-5472.can-17-1143 28951465 PMC6637769

[B59] Zamudio A. V. Dall’agnese A. Henninger J. E. Manteiga J. C. Afeyan L. K. Hannett N. M. (2019). Mediator condensates localize signaling factors to key cell identity genes. *Mol. Cell.* 76 753.e6–766.e6. 31563432 10.1016/j.molcel.2019.08.016 PMC6898777

[B60] Zhang L. Chen S. Wang B. Su Y. Li S. Liu G. (2019). An eight-long noncoding RNA expression signature for colorectal cancer patients’ prognosis. *J. Cell. Biochem.* 120 5636–5643. 10.1002/jcb.27847 30320902

