# Supplementary material for: An Enhancer-Based Analysis Revealed a New Function of Androgen Receptor in Tumor Cell Immune Evasion
Source: Front Genet. 2020 Dec 2;11:595550. doi: 10.3389/fgene.2020.595550 (PMC7738566; doi:10.3389/fgene.2020.595550)
Supplement: Supplementary file 1 [file Image_1.PDF]

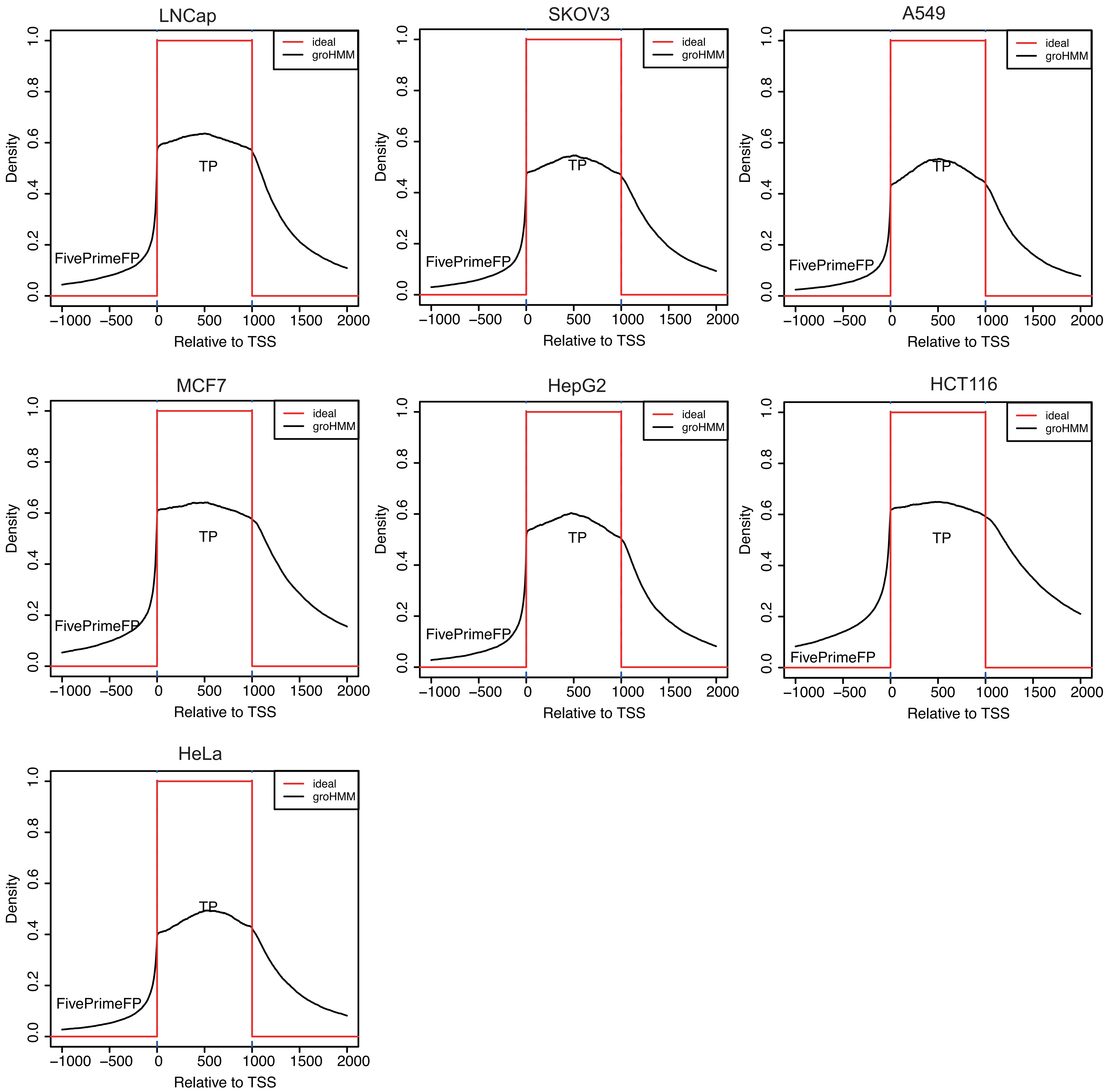

Figure.S1. The performance of groHMM in 'transcribed'region prediction. The y-axis shows the density of reads from GRO-seq mapping to TSS and flanking region. The ideal results (red) have all reads around TSS. The observed results (black) show enrichment of reads on TSS region.
