# Supplementary material for: An Enhancer-Based Analysis Revealed a New Function of Androgen Receptor in Tumor Cell Immune Evasion
Source: Front Genet. 2020 Dec 2;11:595550. doi: 10.3389/fgene.2020.595550 (PMC7738566; doi:10.3389/fgene.2020.595550)
Supplement: Supplementary file 2 [file Image_2.PDF]

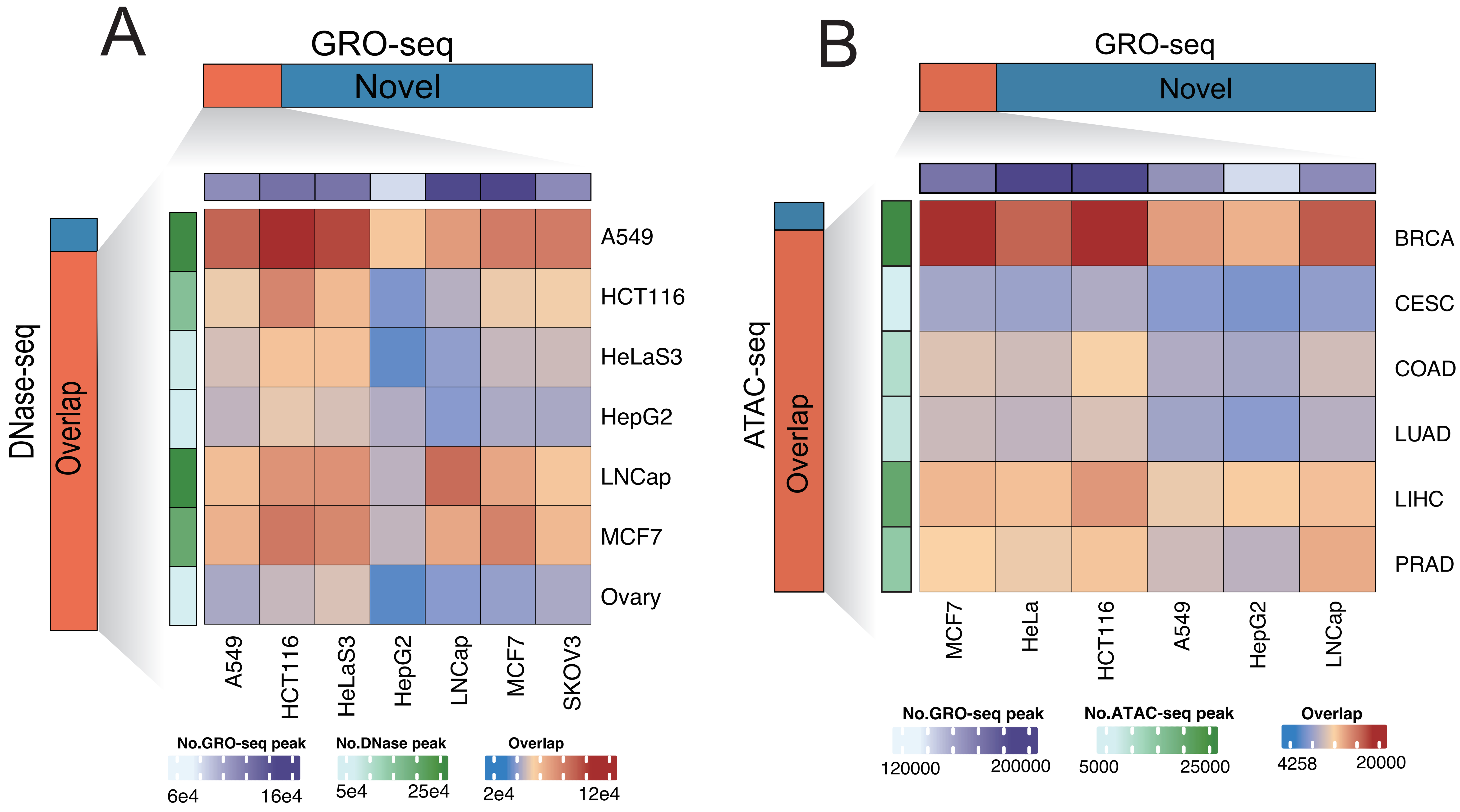

Figure.S2. GRO-seq is sensitive in detecting regions with transcription ability. GRO-seq defined 'transcripts' were compared with DNase-seq peaks (A) and ATAC-seq peaks (B). The outer bar on the left and top of each heatmap show the percentage of DNase-seq peaks/ATAC-seq peaks that can also be covered by GRO-seq (left); and the GRO-seq defined transcripts that can be covered by DNase/ATAC (top). The inner bar on the left and top show the total number of DNase-seq peaks (left) and GRO-seq transcripts (top) in each cancer types. Each cell in the heatmap indicates the number of overlapped regions between two methods in/between certain cancer types.
