# Supplementary material for: An Enhancer-Based Analysis Revealed a New Function of Androgen Receptor in Tumor Cell Immune Evasion
Source: Front Genet. 2020 Dec 2;11:595550. doi: 10.3389/fgene.2020.595550 (PMC7738566; doi:10.3389/fgene.2020.595550)
Supplement: Supplementary file 3 [file Image_3.PDF]

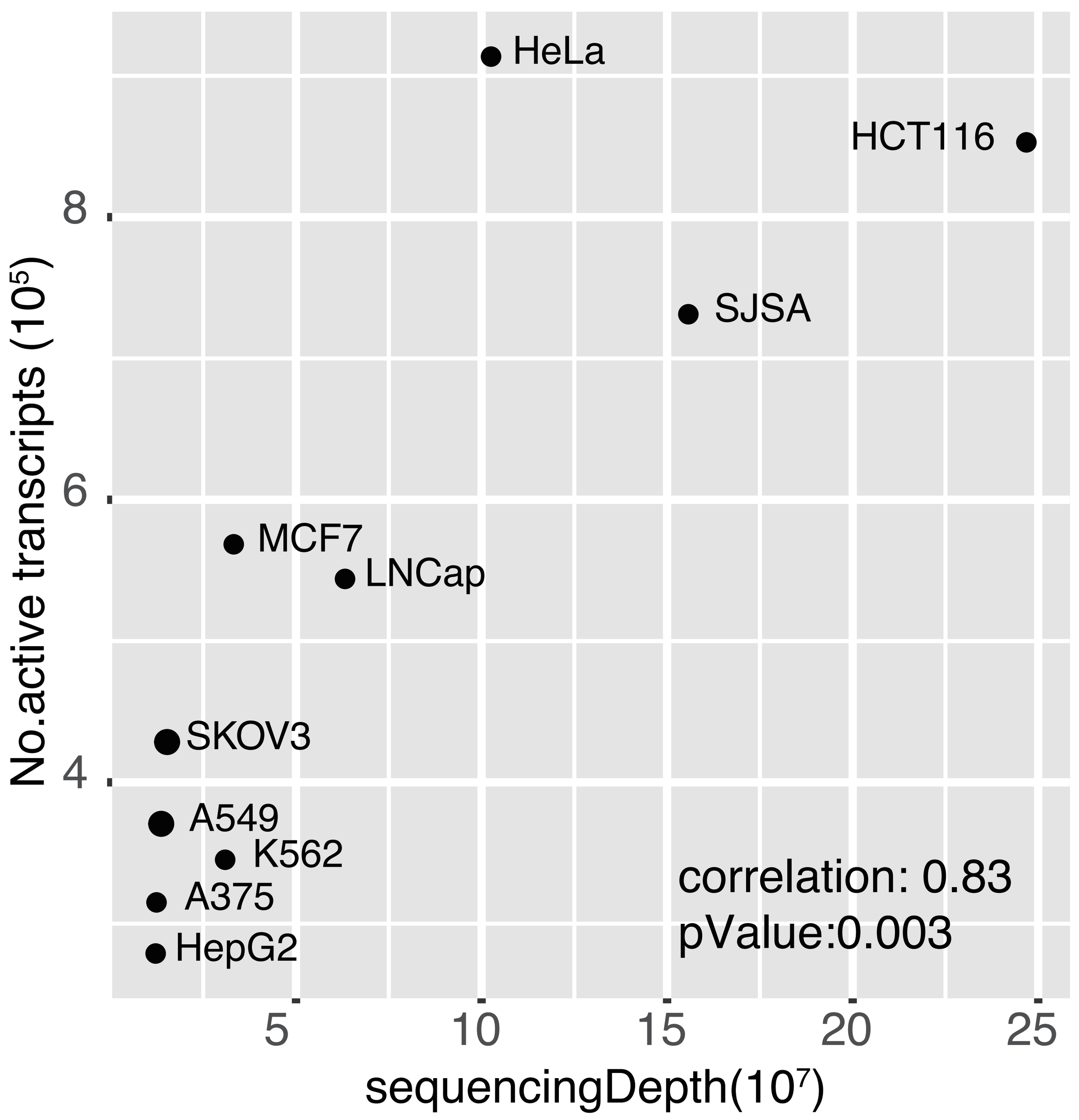


**Figure.S3. The number of transcripts from GRO-seq is related with sequencing depth.** Each cancer cell line was showed as one dot, the y-axis indicates the total number of transcripts predicted from GRO-seq data by groHMM method and x-axis indicates the total number of reads counting from mapping .bam files. The correlation was calculated with Pearson correlation analysis.
