# Supplementary material for: An Enhancer-Based Analysis Revealed a New Function of Androgen Receptor in Tumor Cell Immune Evasion
Source: Front Genet. 2020 Dec 2;11:595550. doi: 10.3389/fgene.2020.595550 (PMC7738566; doi:10.3389/fgene.2020.595550)
Supplement: Supplementary file 4 [file Image_4.PDF]

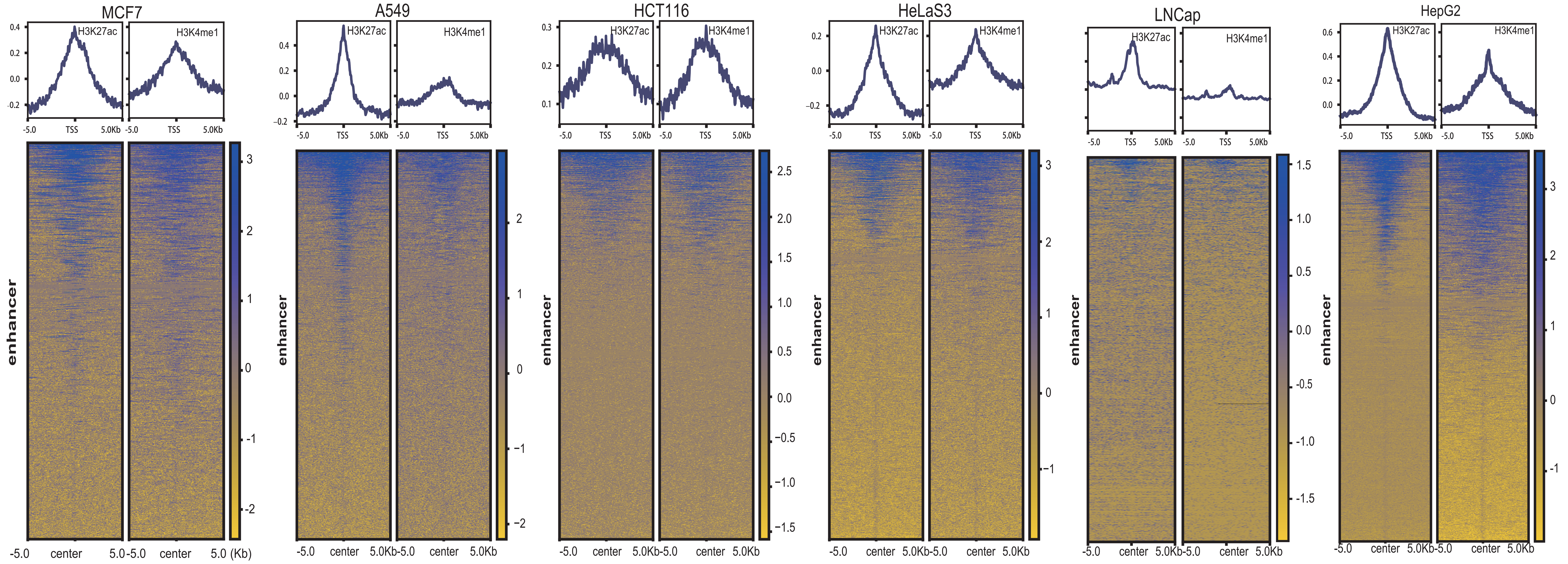

Figure.S4. GRO-seq defined enhancers were enriched with enhancer markers. ChIP-seq signals of H3K27ac and H3K4me1 show higher in the center of GRO-seq defined enhancer loci. Each line in the heatmap indicates one enhancer locus, and the signals were calculated with Deeptools.
