# Supplementary material for: An Enhancer-Based Analysis Revealed a New Function of Androgen Receptor in Tumor Cell Immune Evasion
Source: Front Genet. 2020 Dec 2;11:595550. doi: 10.3389/fgene.2020.595550 (PMC7738566; doi:10.3389/fgene.2020.595550)
Supplement: Supplementary file 5 [file Image_5.PDF]

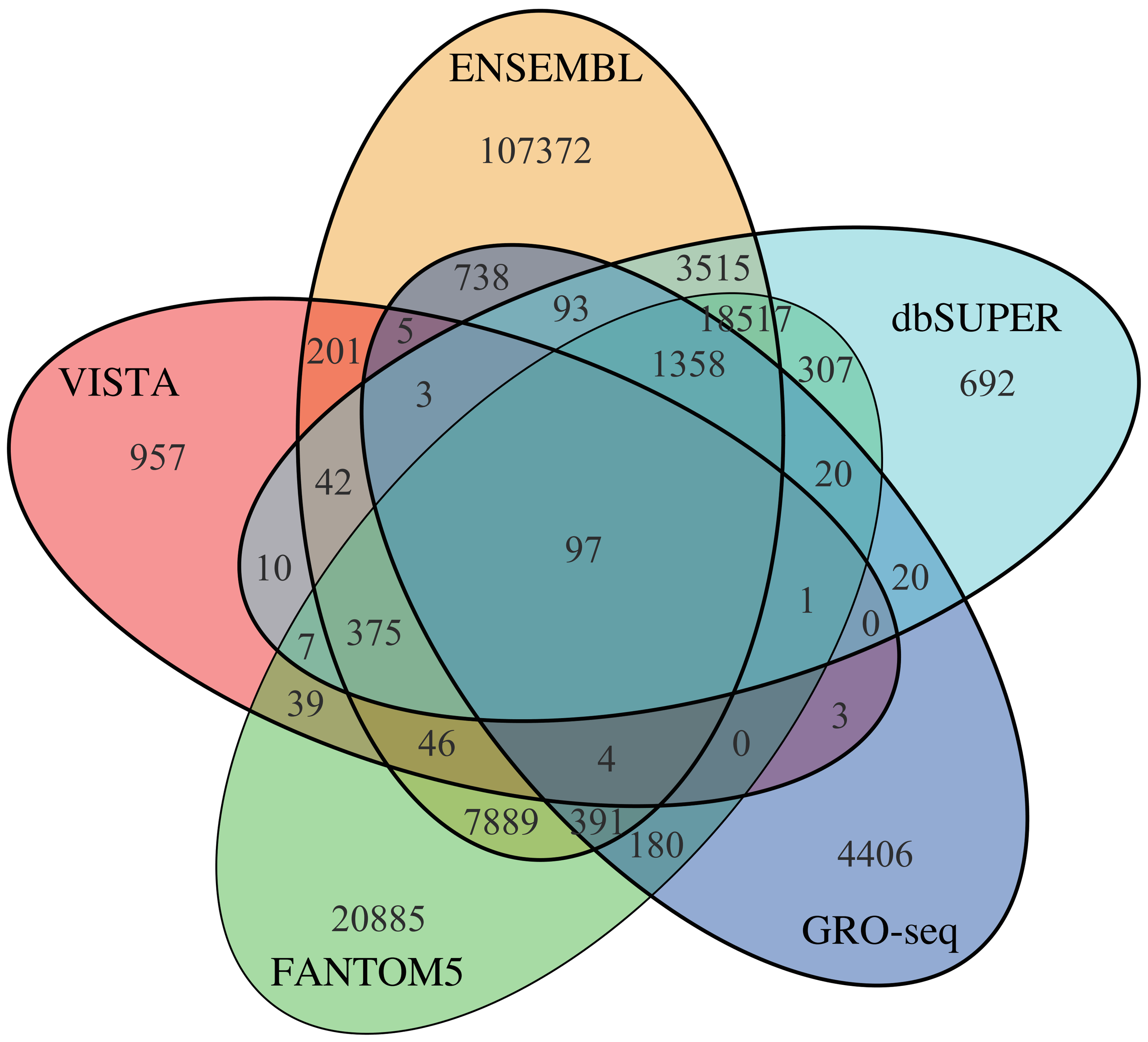

Figure.S5. Venn plot shows the overlap among GRO-seq defined enhancer, and the enhancer sets from ENSEMBL, FANTOM5, dbSUPER and VISTA.
