# Supplementary material for: An Enhancer-Based Analysis Revealed a New Function of Androgen Receptor in Tumor Cell Immune Evasion
Source: Front Genet. 2020 Dec 2;11:595550. doi: 10.3389/fgene.2020.595550 (PMC7738566; doi:10.3389/fgene.2020.595550)
Supplement: Supplementary file 6 [file Image_6.PDF]

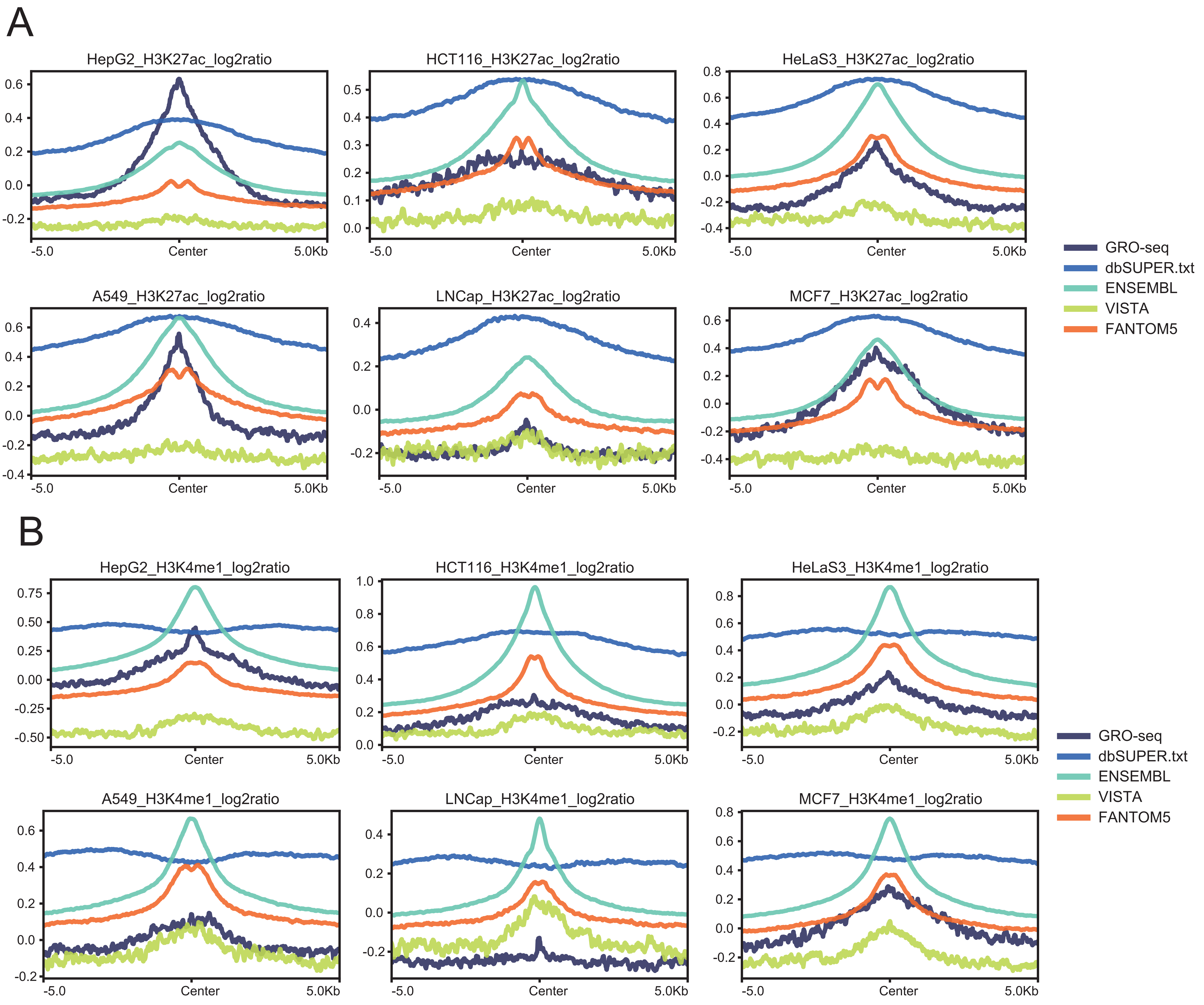

Figure.S6. Comparing the signal of H3K27ac (A) and H3K4me1 (B) among GRO-seq enhancers and enhancers from other databases. The signals were calculated on enhancer center and its flanking 5kb region.
