# Supplementary material for: An Enhancer-Based Analysis Revealed a New Function of Androgen Receptor in Tumor Cell Immune Evasion
Source: Front Genet. 2020 Dec 2;11:595550. doi: 10.3389/fgene.2020.595550 (PMC7738566; doi:10.3389/fgene.2020.595550)
Supplement: Supplementary file 7 [file Image_7.PDF]

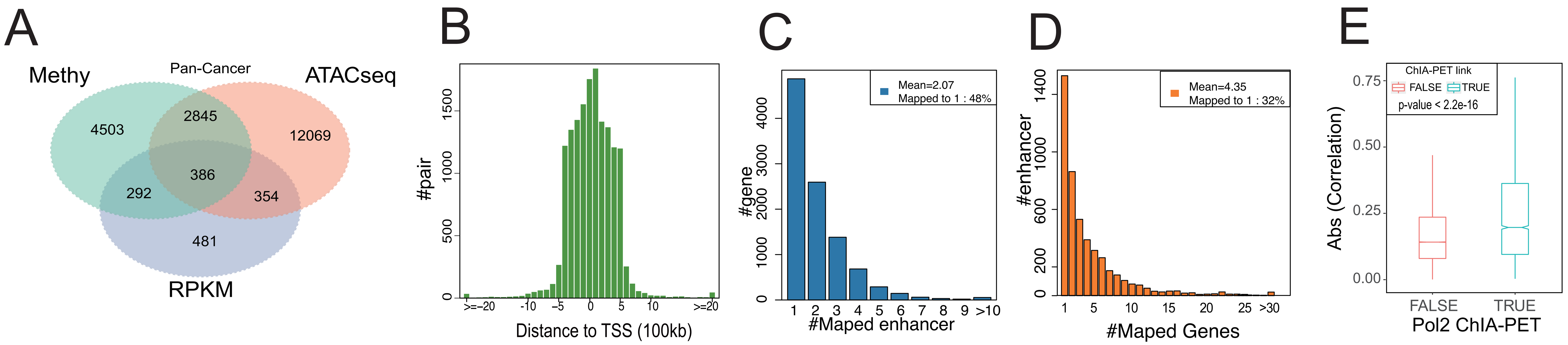


**Figure.S7.** (A) shows the overlap of EG pairs predicted by ATAC-seq, DNA methylation and RNA-seq data. The prediction is based on correlation between enhancer activity which is measured by ATAC-seq/methylation/RNA-seq and gene expression measured by RNA-seq. (B) the height of bars showed the number of EG pairs with certain distance between enhancer and target genes. (C) The height of bars showed the number of genes which is targeted by certain number of enhancers. (D) the height of bars showed the number of genes which is targeted by certain number of genes. (E) The figure compared the correlation between EG pairs which connection is supported by MCF7 Pol II ChIA-PET (blue) and those without support (red).
