# Supplementary material for: An Enhancer-Based Analysis Revealed a New Function of Androgen Receptor in Tumor Cell Immune Evasion
Source: Front Genet. 2020 Dec 2;11:595550. doi: 10.3389/fgene.2020.595550 (PMC7738566; doi:10.3389/fgene.2020.595550)
Supplement: Supplementary file 8 [file Image_8.PDF]

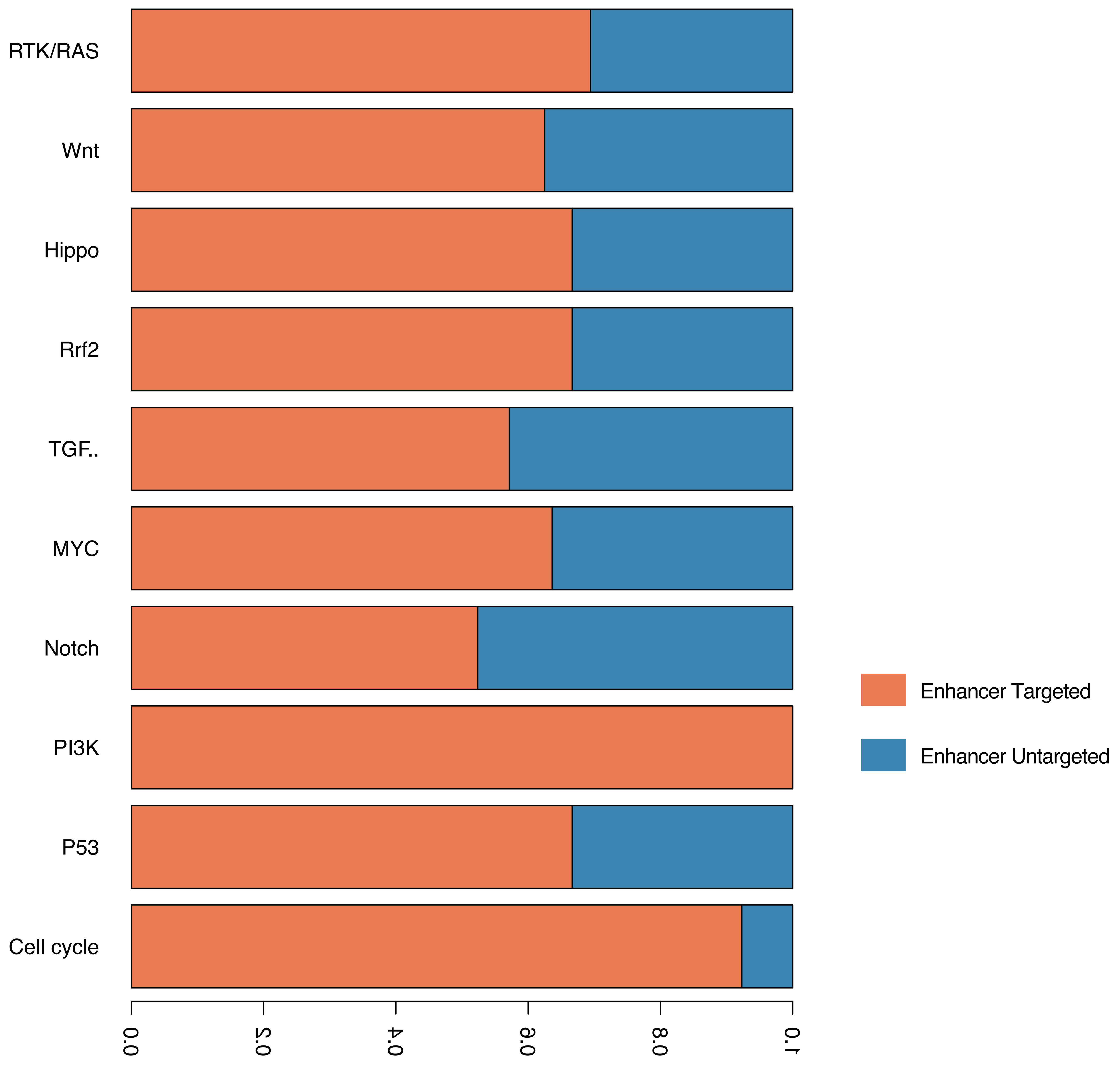

Figure.S8. Most of cancer signaling pathway genes were regulated by enhancer. The barplot show the proportion of genes that correlated with enhancer activity for each cancer signaling pathway.
