# Supplementary material for: An Enhancer-Based Analysis Revealed a New Function of Androgen Receptor in Tumor Cell Immune Evasion
Source: Front Genet. 2020 Dec 2;11:595550. doi: 10.3389/fgene.2020.595550 (PMC7738566; doi:10.3389/fgene.2020.595550)
Supplement: Supplementary file 9 [file Image_9.PDF]

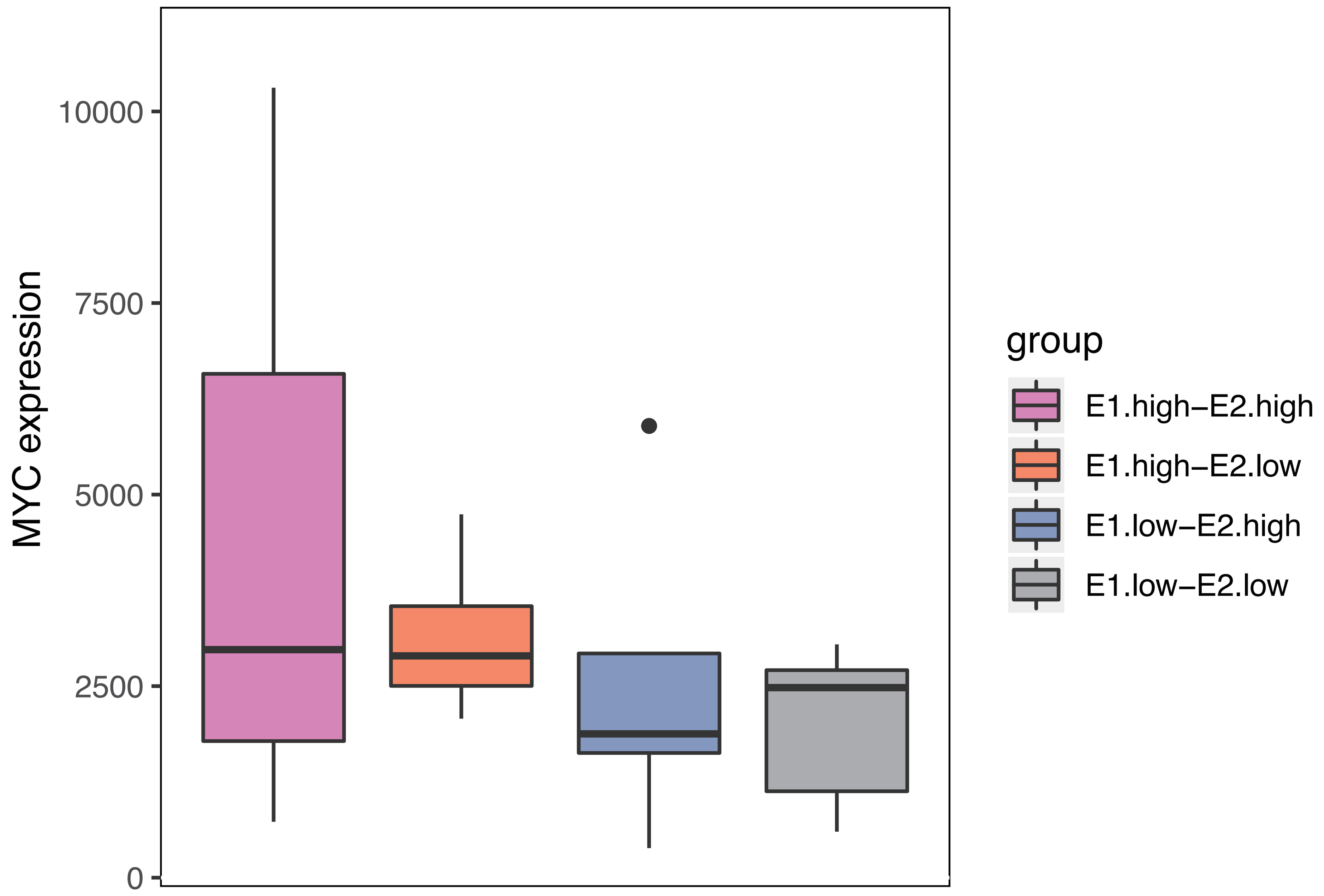

	Df	Sum Sq	Mean Sq	F value	Pr(>F)
E1	1	66225046	66225046	31.991	2.85e-05
E2	1	322300	322300	0.156	0.69806
E1:E2	1	20706730	20706730	10.003	0.00569
Residuals	17	35191412	2070083		

Figure.S9. E1 contribute to MYC activation more than E2 does in STAD. To plot the boxplot, the STAD tumor samples were divided into 4 groups according to E1 and E2 ATAC-seq signals: 'E1.high-E2.high' group, with both E1 and E2 signals higher than median value; 'E1.high-E2.low' group, with E1 signal higher than median but E2 signal lower than median; 'E1.low-E2.high' group, with E1 signal lower than median but E2 signal higher than median; 'E1.low-E2.low' group, with both E1 and E2 signal lower than median. The down panel shows the results from one-way and multivariate analysis of variance. The groups with E1 high activity show significant (Pr(>F)>0.05) higher MYC expression than other samples.
