# Supplementary material for: An Enhancer-Based Analysis Revealed a New Function of Androgen Receptor in Tumor Cell Immune Evasion
Source: Front Genet. 2020 Dec 2;11:595550. doi: 10.3389/fgene.2020.595550 (PMC7738566; doi:10.3389/fgene.2020.595550)
Supplement: Supplementary file 10 [file Image_10.PDF]

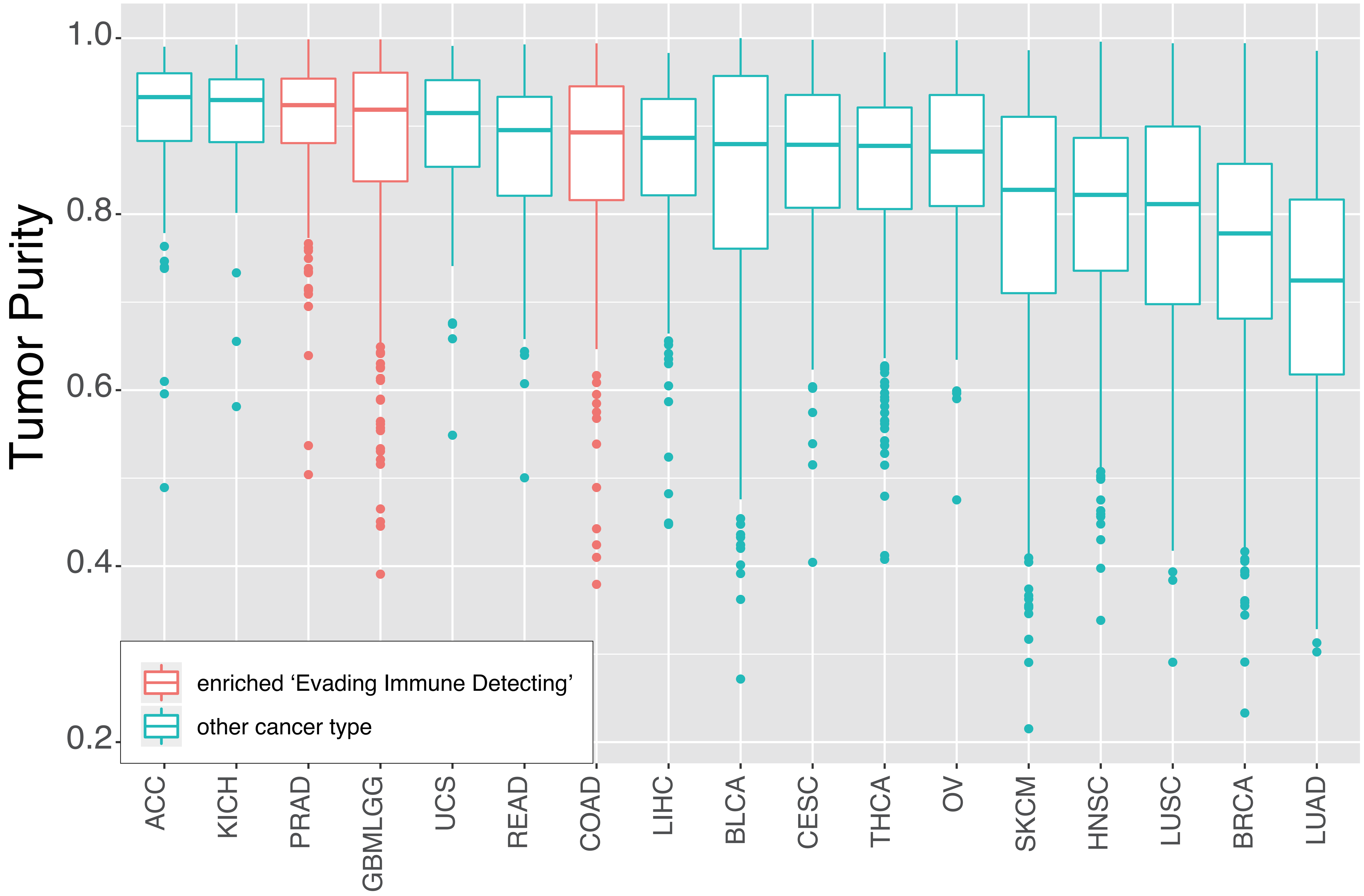

Figure.S10. The tumors which enhancer contribute to 'Evading Immune Detection' show relative higher tumor purity. The tumor purity data was from TCGAbiolinks. The tumor which enhancer targets enriched in EID were labeled in red.
