# Supplementary material for: An Enhancer-Based Analysis Revealed a New Function of Androgen Receptor in Tumor Cell Immune Evasion
Source: Front Genet. 2020 Dec 2;11:595550. doi: 10.3389/fgene.2020.595550 (PMC7738566; doi:10.3389/fgene.2020.595550)
Supplement: Supplementary file 11 [file Image_11.PDF]

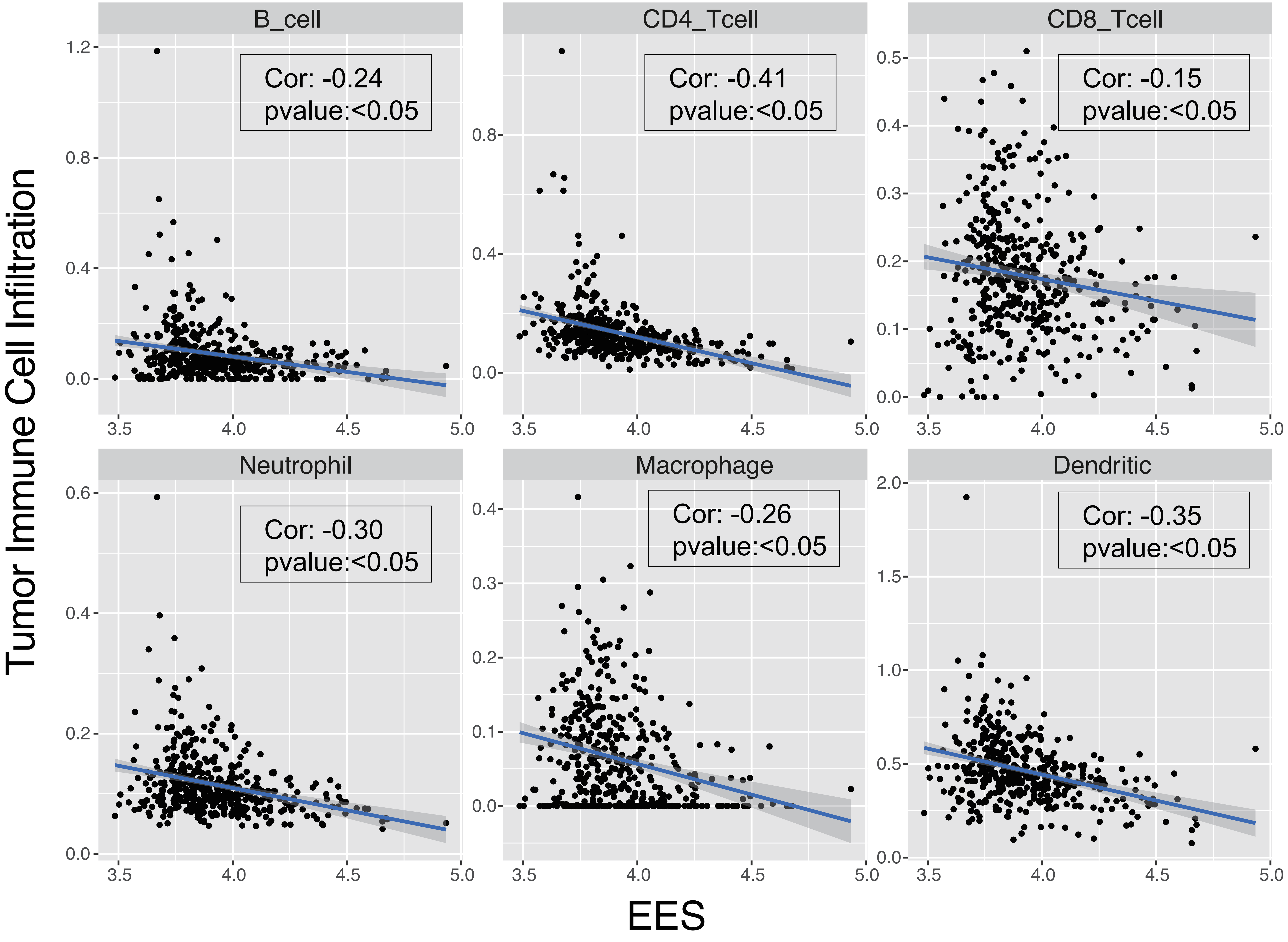

Figure.S11. Enhancer Effect Score (EES) is related with immune cell infiltration in prostate cancer. The infiltration information for different immune cells in each prostate cancer samples were obtained from TIMER database. The correlation was calculated by Pearson Correlation analysis.
