# Supplementary figures and images for: An Enhancer-Based Analysis Revealed a New Function of Androgen Receptor in Tumor Cell Immune Evasion
Source: Front Genet. 2020 Dec 2;11:595550. doi: 10.3389/fgene.2020.595550 (PMC7738566; doi:10.3389/fgene.2020.595550)

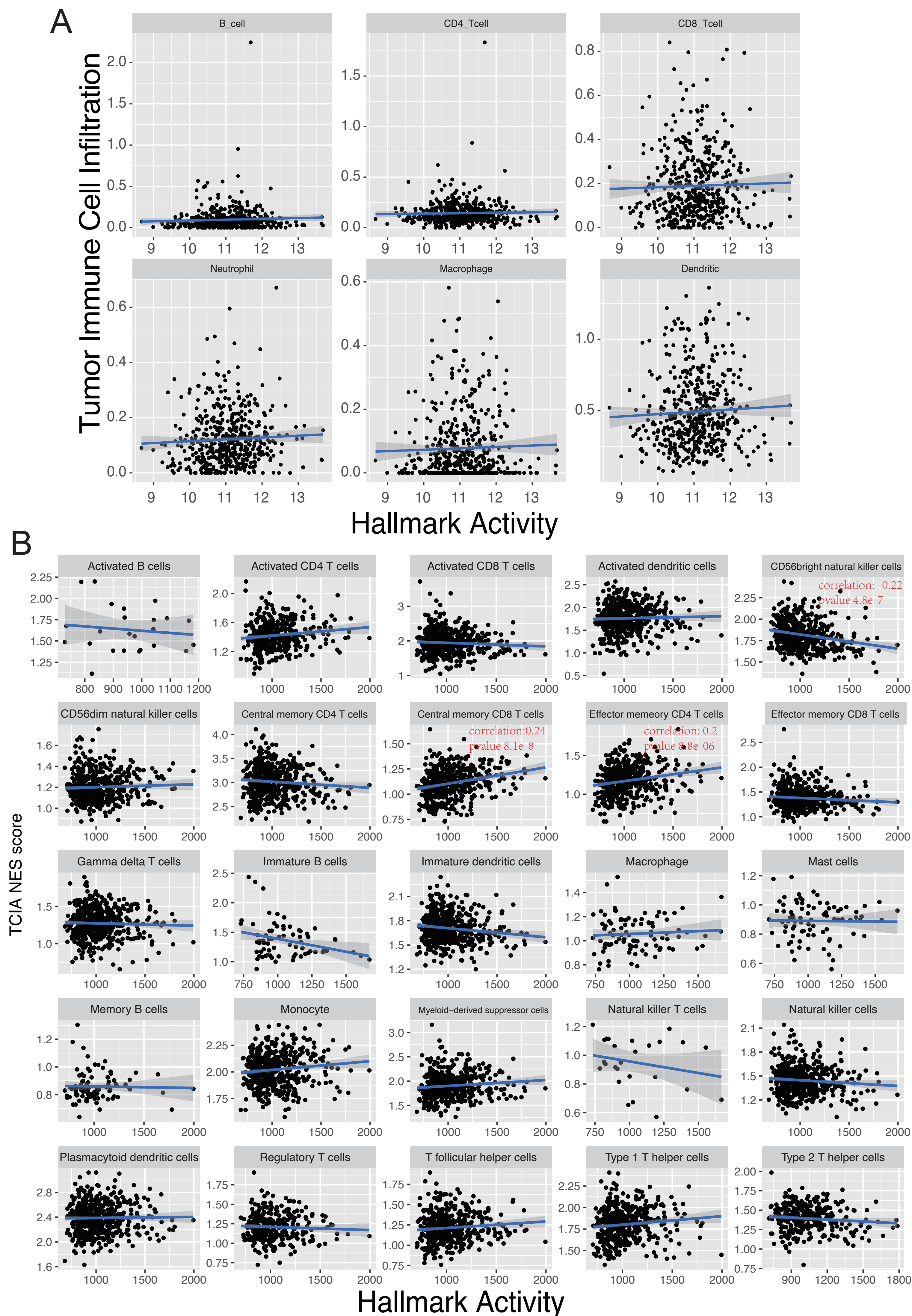

Supplement: Supplementary file 12 [file Image_12.PDF]
