# Supplementary material for: An Enhancer-Based Analysis Revealed a New Function of Androgen Receptor in Tumor Cell Immune Evasion
Source: Front Genet. 2020 Dec 2;11:595550. doi: 10.3389/fgene.2020.595550 (PMC7738566; doi:10.3389/fgene.2020.595550)
Supplement: Supplementary file 13 [file Image_13.PDF]

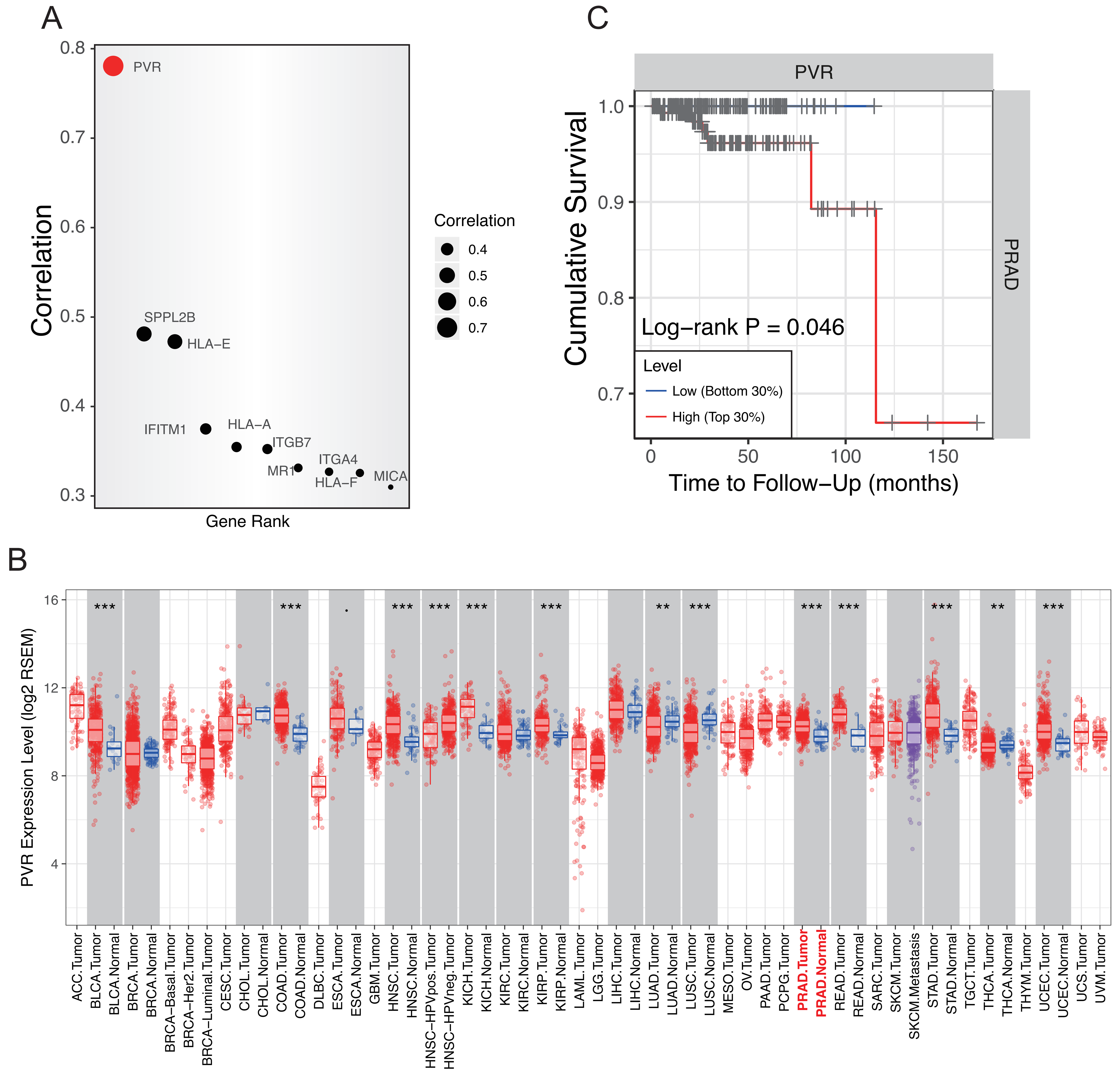

Figure.S13. PVR contribute to gain of EID hallmark in prostate cancer. (A) The EG pairs belong to EID hallmark was shown as dot in the figure. The gene was ranking according to their correlation. The EG pair with highest correlation was labeled in red. (B) The PVR gene expression across all the tumor and normal samples in TCGA datasets. The figure was downloaded from TIMER database. (C) PVR expression was related with poor survival in prostate cancer. TCGA prostate tumor samples were grouped into two groups according to the PVR expression, the samples with lower PVR expression than 30% percentile were considered as low group and the ones with higher PVR expression than 70% percentile were considered as high group. The figure was download from TIMER database.
