# Supplementary material for: An Enhancer-Based Analysis Revealed a New Function of Androgen Receptor in Tumor Cell Immune Evasion
Source: Front Genet. 2020 Dec 2;11:595550. doi: 10.3389/fgene.2020.595550 (PMC7738566; doi:10.3389/fgene.2020.595550)
Supplement: Supplementary file 14 [file Image_14.PDF]

Figure.S14

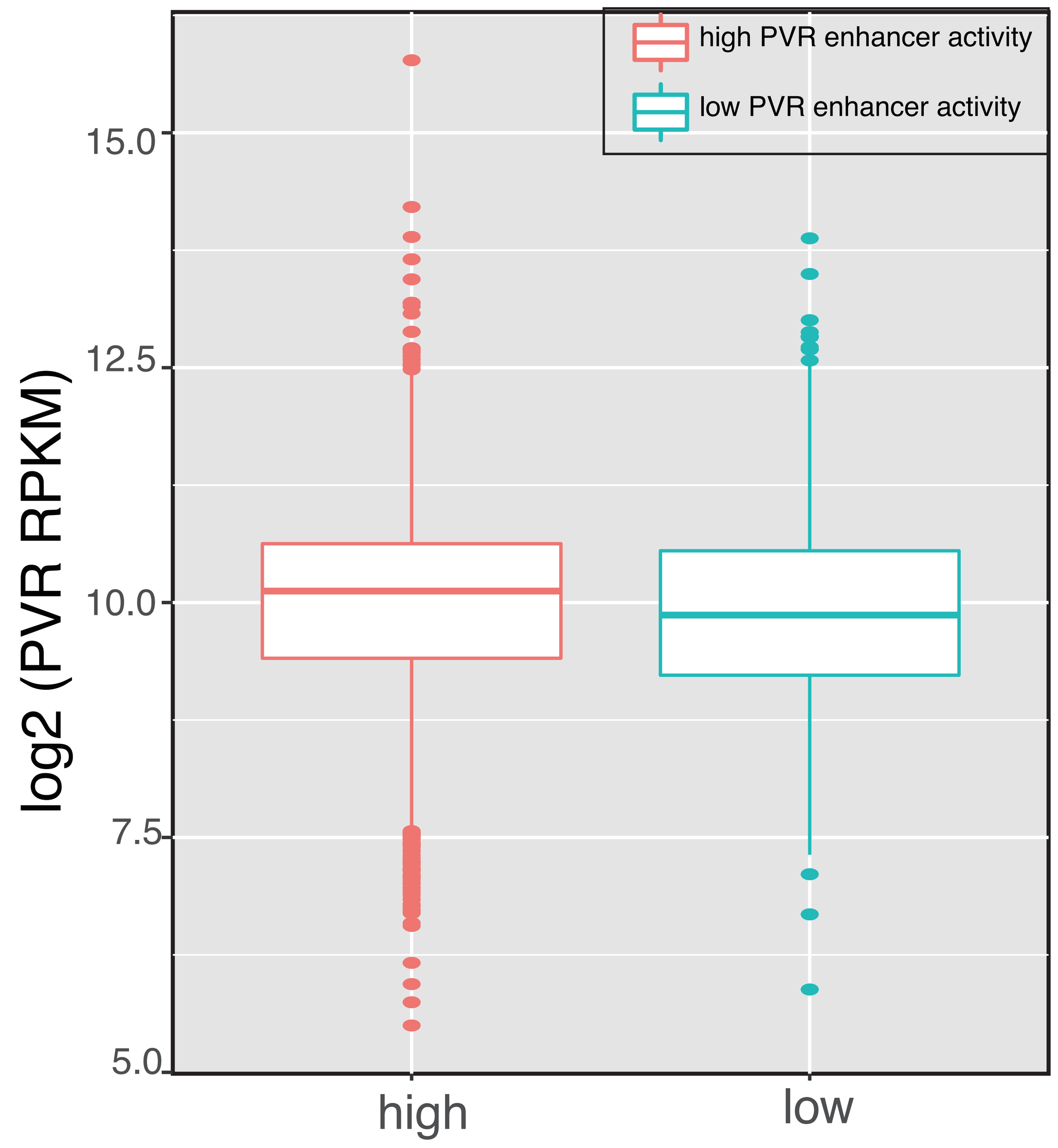

Figure.S16

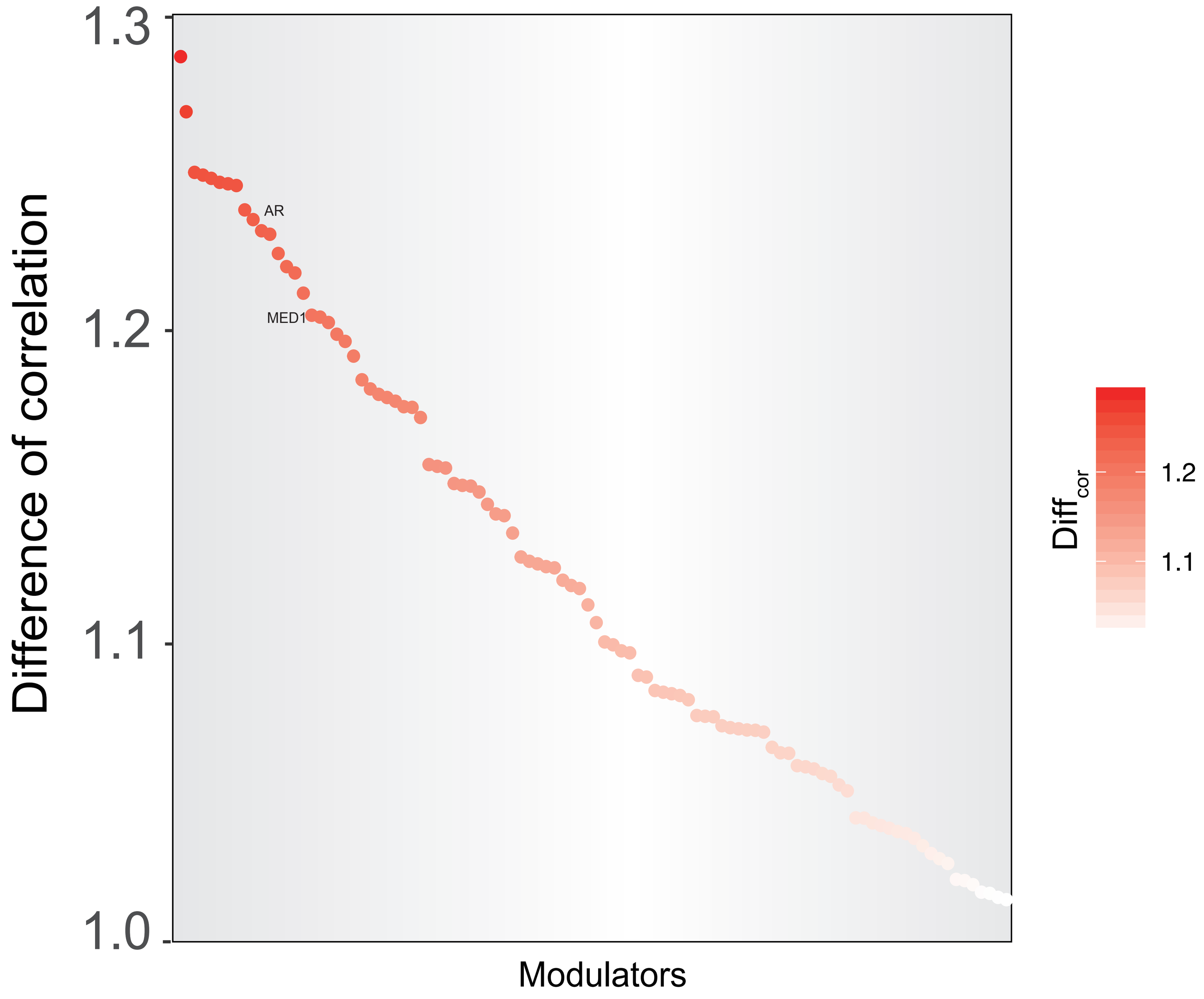

Figure. S15

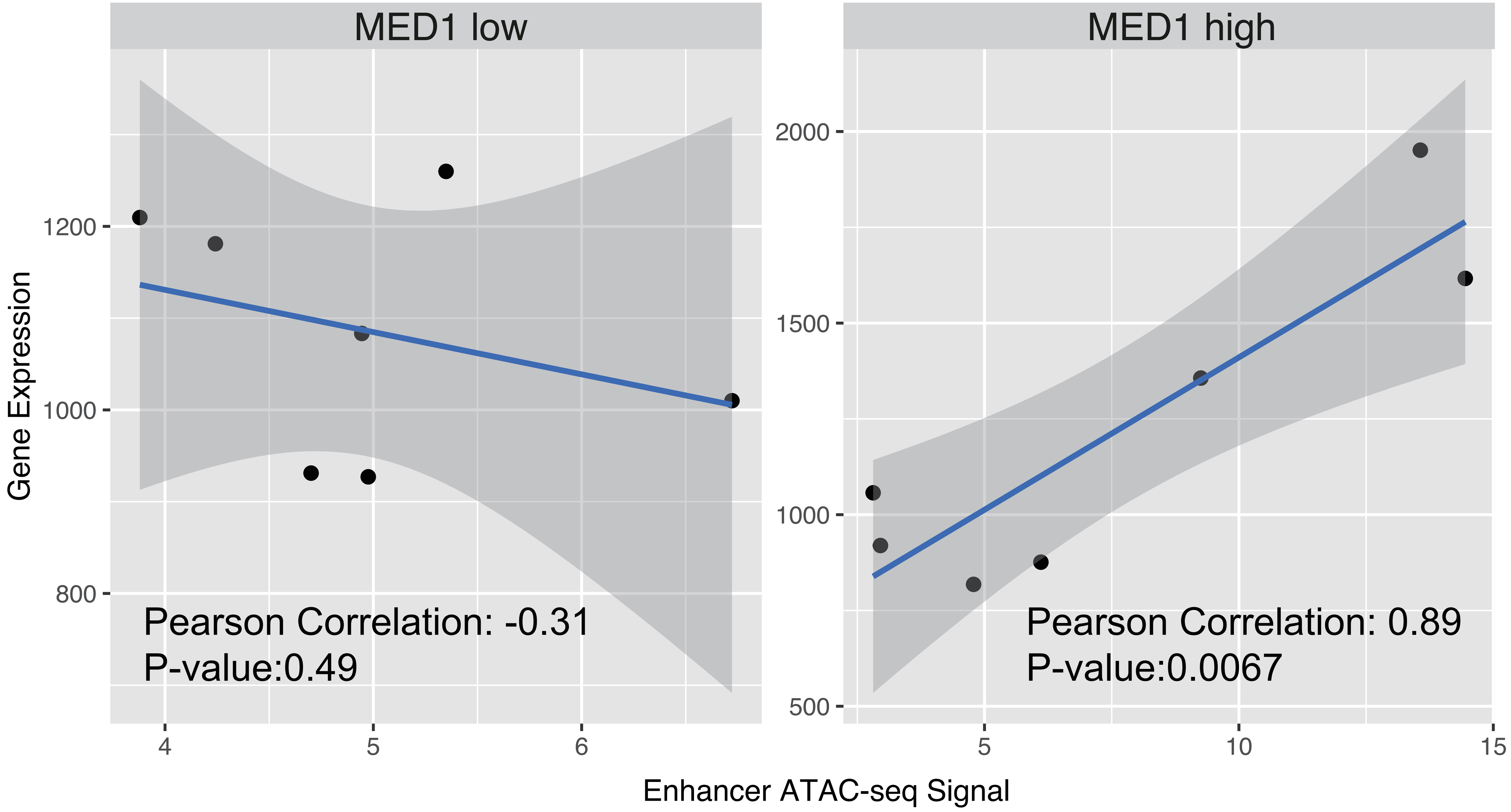

Figure.S14. Tumors with low PVR enhancer activity show lower PVR expression accordingly. All cancer types in TCGA were separated into two groups according to the average enhancer activity value (ATAC-seq signal). PVR expression was higher in high group than in low group with T-test p-value as 0.0007471

Figure.S15. MED1 mediated enhancer connection with PVR. The prostate samples were divided into two group according to the MED1 expression. The samples with lower MED1 expression than the first quartile was considered as low group (left) and the samples with higher MED1 expression than the last quartile was considered as high group (right). The correlation was calculated between enhancer ATAC-seq signals and PVR expression in each of the group.

Figure.S16. Top 100 modulators were shown as red dot according to the difference correlation value between high group and low group (see method). AR and MED1 were labeled in the figure as they are in hub position of network in Figure.6.C.
