# Supplementary material for: An Enhancer-Based Analysis Revealed a New Function of Androgen Receptor in Tumor Cell Immune Evasion
Source: Front Genet. 2020 Dec 2;11:595550. doi: 10.3389/fgene.2020.595550 (PMC7738566; doi:10.3389/fgene.2020.595550)
Supplement: Supplementary file 15 [file Image_15.PDF]

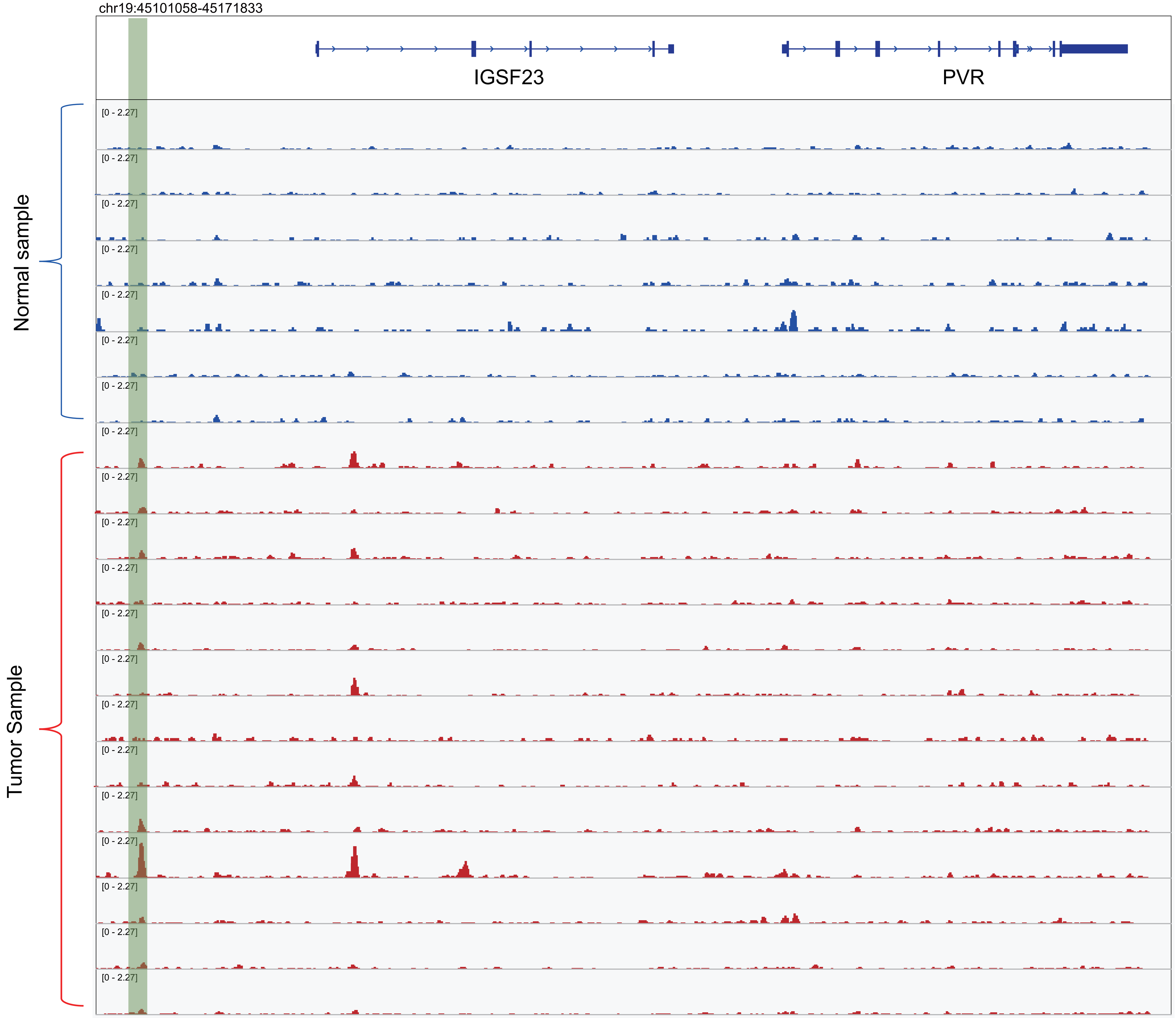

Figure.S17. AR ChIP-seq results on PVR and enhancer locus. The region under green shadow was predicted PVR enhancer region. The blue track was AR ChIP-seq from prostate normal samples and red track was AR ChIP-seq from prostate tumor samples.
